# SNP-array lesions in core binding factor acute myeloid leukemia

**DOI:** 10.18632/oncotarget.24031

**Published:** 2018-01-08

**Authors:** Nicolas Duployez, Elise Boudry-Labis, Christophe Roumier, Nicolas Boissel, Arnaud Petit, Sandrine Geffroy, Nathalie Helevaut, Karine Celli-Lebras, Christine Terré, Odile Fenneteau, Wendy Cuccuini, Isabelle Luquet, Hélène Lapillonne, Catherine Lacombe, Pascale Cornillet, Norbert Ifrah, Hervé Dombret, Guy Leverger, Eric Jourdan, Claude Preudhomme

**Affiliations:** ^1^ CHU Lille, Laboratory of Hematology, Biology and Pathology Center, Lille, France; ^2^ Univ. Lille, INSERM, UMR-S 1172, JPARC, Lille, France; ^3^ CHU Lille, Institute of Medical Genetics, Jeanne de Flandre Hospital, Lille, France; ^4^ APHP, Department of Hematology, Saint Louis Hospital, Paris, France; ^5^ APHP, Department of Pediatric Hematology and Oncology, GH HUEP, Trousseau Hospital, Paris, France; ^6^ Sorbonne Universites, UPMC Univ Paris 06, UMR-S 938, CDR Saint-Antoine, GRC n°07, GRC MyPAC, Paris, France; ^7^ CH Versailles, Department of Genetics, Le Chesnay, France; ^8^ APHP, Laboratory of Hematology, Robert Debré University Hospital, Paris, France; ^9^ APHP, Department of Cytogenetics, Saint Louis Hospital, Paris, France; ^10^ CHU Toulouse, Laboratory of Hematology, Toulouse, France; ^11^ APHP, Laboratory of Hematology, GH HUEP, Trousseau Hospital, Paris, France; ^12^ APHP, Goelamsthèque, Cochin Hospital, Paris, France; ^13^ CHU Reims, Laboratory of Hematology, Reims, France; ^14^ CHU Angers, INSERM CRCINA, Department of Hematology, Angers, France; ^15^ CHU Nîmes, Department of Hematology, Nîmes, France

**Keywords:** acute myeloid leukemia, core binding factor, SNP-array, sequencing

## Abstract

Acute myeloid leukemia (AML) with t(8;21) and inv(16), together referred as core binding factor (CBF)-AML, are recognized as unique entities. Both rearrangements share a common pathophysiology, the disruption of the CBF, and a relatively good prognosis. Experiments have demonstrated that CBF rearrangements were insufficient to induce leukemia, implying the existence of cooperating events. To explore these aberrations, we performed single nucleotide polymorphism (SNP)-array in a well-annotated cohort of 198 patients with CBF-AML. Excluding breakpoint-associated lesions, the most frequent events included loss of a sex chromosome (53%), deletions at 9q21 (12%) and 7q36 (9%) in patients with t(8;21) compared with trisomy 22 (13%), trisomy 8 (10%) and 7q36 deletions (12%) in patients with inv(16). SNP-array revealed novel recurrent genetic alterations likely to be involved in CBF-AML leukemogenesis. *ZBTB7A* mutations (20% of t(8;21)-AML) were shown to be a target of copy-neutral losses of heterozygosity (CN-LOH) at chromosome 19p. *FOXP1* focal deletions were identified in 5% of inv(16)-AML while sequence analysis revealed that 2% carried *FOXP1* truncating mutations. Finally, *CCDC26* disruption was found in both subtypes (4.5% of the whole cohort) and possibly highlighted a new lesion associated with aberrant tyrosine kinase signaling in this particular subtype of leukemia.

## INTRODUCTION

CBF-AML, including AML with t(8;21) and AML with inv(16)/t(16;16), accounts for approximately 25% of pediatric and 15% of adult *de novo* AML patients. Compared to other AML subsets, CBF-AML is considered to have a good prognosis. Both alterations result in disruption of genes encoding subunits of the CBF (*i.e*. *RUNX1* and *CBFB*), a heterodimeric transcription factor complex required for normal hematopoiesis [1]. Importantly, experiences from murine models [2], as well as the existence of preleukemic cells harboring a CBF rearrangement in healthy individuals [3, 4], have demonstrated that CBF disruption is insufficient to induce leukemia. CBF-AML is therefore considered as a model of multistep pathogenesis. Evidences supporting this model have been generated by the high frequency of cooperative events at time of diagnosis. Notably, mutations in genes encoding tyrosine kinase pathways effectors (especially *KIT*, *FLT3* and *RAS* mutations) are found in up to 80% of CBF-AML patients [5–7]. Additional chromosomal aberrations are detected in approximately 70% of patients with t(8;21)-AML and 40% of patients with inv(16)-AML by conventional karyotype [5, 8–10]. These aberrations are nonrandom events and some of them are extremely rare in non-CBF-AML. In this context, the identification of recurrent events involved in CBF-AML pathophysiology and heterogeneity remains of great interest. We report here the SNP-array profiling of a large and well-annotated cohort of pediatric and adult patients with CBF-AML and the identification of new recurrent lesions in this particular subtype of leukemia.

## RESULTS

### CBF-AML genomes are characterized by a limited number of SNP-array-lesions

SNP-array analysis of 116 t(8;21)-AML and 82 inv(16)-AML revealed a total of 319 lesions, including 277 copy-number abnormalities (CNAs; 187 losses and 90 gains; median size: 26.1 Mb [range: 26 kb-155.1 Mb]) and 42 CN-LOH (Supplementary Table 1). Overall, 97 (84%) patients with t(8;21)-AML and 55 (64%) patients with inv(16)-AML had at least one genomic aberration (CNA and/or CN-LOH). There was no significant difference in the number of lesions between adult and pediatric patients (Supplementary Table 2) arguing for similar diseases as previously described [6]. Recurrent focal lesions associated with t(8;21) and inv(16) breakpoints were common events, occurring in 27 (14%) CBF-AML cases especially in the inv(16) subtype (22% *vs.* 7%, p=0.005). Considering them as part of the primary event, t(8;21) or inv(16), breakpoint-associated lesions (accounting for 41 of the 319 identified lesions) were excluded for subsequent descriptions. Finally, CBF-AML genomes exhibited a mean of 1.40 SNP-array aberrations per case (range: 0-7)(Table 1). CNAs were more numerous in t(8;21)-AML than in inv(16)-AML, mostly due to genomic deletions (0.98 *vs.* 0.44 losses/case respectively; p<0.001). Neither the presence of SNP-array lesions nor the number of lesions was a predictor of outcome (Supplementary Figure 1).

**Table 1:** Mean number of SNP-array lesions per CBF AML case according to genetic subtype

	CBF AML	t(8;21) AML	inv(16) AML	p-value	
Patients, n	198	116	82		
Number of CNAs^†^, mean (range)	1.19 (0-6)	1.35 (0-5)	0.96 (0-6)	0.004	^*^
Gains^†^, mean (range)	0.43 (0-4)	0.37 (0-4)	0.52 (0-3)	0.084	
Losses^†^, mean (range)	0.76 (0-4)	0.98 (0-3)	0.44 (0-4)	<0.001	^*^
Number of CN-LOH^†^, mean (range)	0.21 (0-6)	0.17 (0-6)	0.27 (0-4)	0.372	
Breakpoint lesions, mean (range)	0.21 (0-2)	0.09 (0-2)	0.38 (0-2)	0.002	^*^
Total CNAs/CN-LOH^†^, mean (range)	1.40 (0-7)	1.53 (0-7)	1.23 (0-7)	0.020	^*^

CNA: copy number abnormality; CN-LOH: copy neutral-loss of heterozygosity.

^†^ excluding breakpoint-associated lesions.

### SNP-array karyotyping in CBF-AML shows nonrandom copy number changes

**Figure 1:**
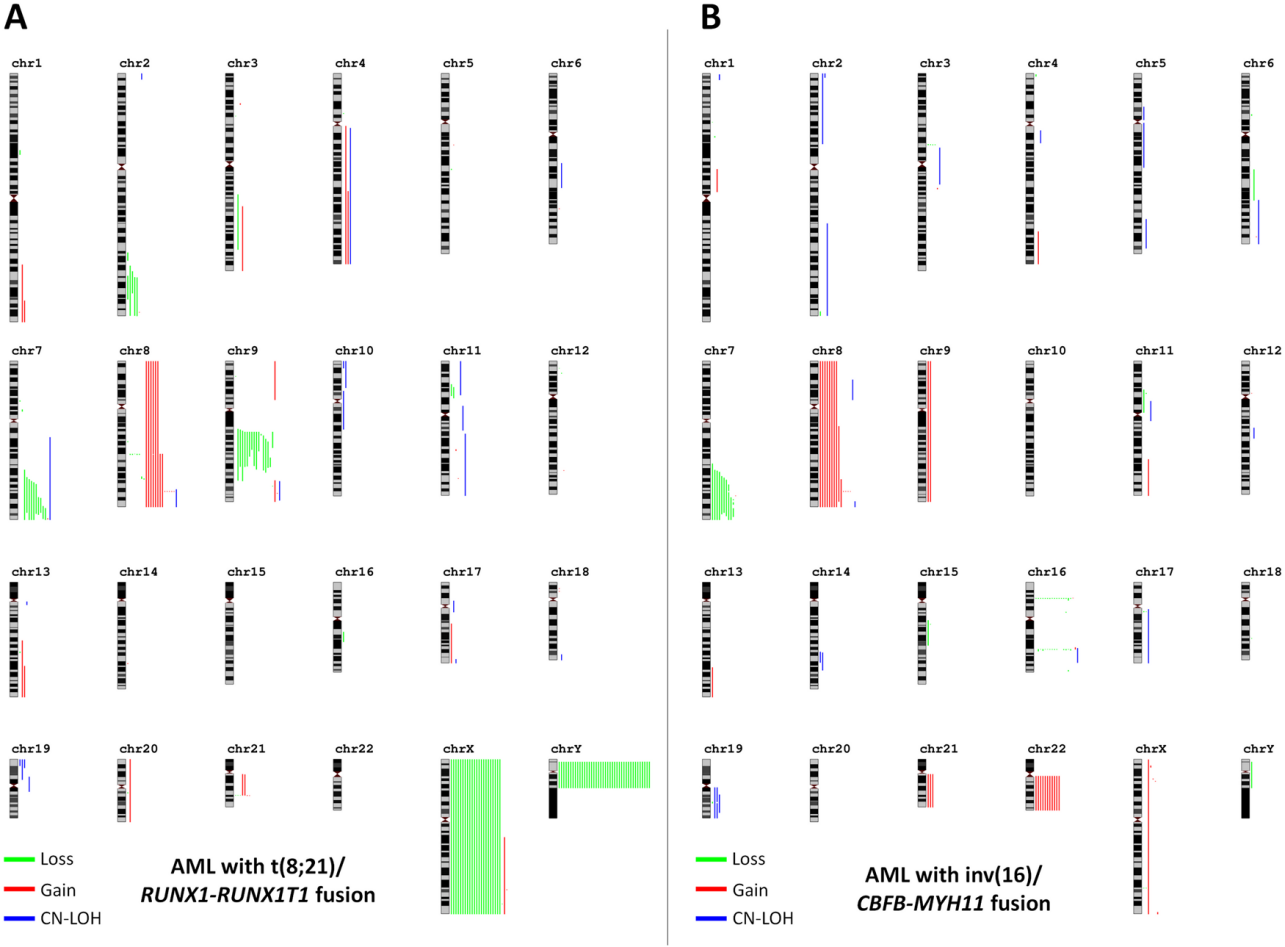
Karyograms of detected SNP-array lesions by genetic subtype Each vertical line represents 1 event in 1 patient. Gains are in red, losses in green and CN-LOH in blue. Part **(A)** shows cases with t(8;21)-AML and part **(B)** shows cases with inv(16)-AML. Schematic representations were obtained using the Genomic Recurrent Event ViEwer (GREVE) web tool (http://www.well.ox.ac.uk/GREVE) [11].

Recurrent CNAs are listed in Table 2. Considering lesions that are non-associated with breakpoints, a large proportion of detected CNAs were broad aberrations or involved whole chromosomes (Figure 1) [11]. Most of them appeared to be nonrandom events and are usually seen by conventional karyotype [1]. Among t(8;21)-AML patients, loss of a sex chromosome (LOS) was by far the most common event, occurring in 62 (53%) patients, followed by interstitial deletion of the long arm of chromosome 9 [del(9q)] in 15 (13%) patients (p<0.001). Both aberrations were virtually absent among inv(16)-AML patients. All but one case with del(9q) shared a minimal deleted region (MRD) of 6.1Mb containing 19 genes (Supplementary Figure 2) in which *TLE1* and *TLE4* have been the most studied [12, 13]. By contrast, +22 was restricted to inv(16)-AML and occurred in 11 (13%) patients. Trisomy 8 and interstitial deletion of the long arm of chromosome 7 [del(7q)] were found in both genetic subtypes. Trisomy 8 was observed in 8 (10%) cases with inv(16) and 6 (5%) cases with t(8;21). Gain of the long arm of chromosome 8 (+8q) was seen is 2 additional cases with inv(16) (2 other cases with t(8;21) had +8q related to the rarely described duplication of the derivate chromosome der(21) t(8;21) [14]; Supplementary Figure 3). Del(7q) was found in 20 (10%) patients, including 10 (9%) cases with t(8;21) and 10 (12%) cases with inv(16). All cases with del(7q), whatever their genetic subtype t(8;21) or inv(16), shared a MDR of 4.2 Mb containing 71 genes in which the 2 epigenetics-related genes *EZH2* and *KMT2C* (*MLL3*) were the more relevant and have already been studied by others [15–17] (Supplementary Figure 4). Overall, we did not find any association between these recurrent genetic aberrations and clinical outcome (Supplementary Figure 5). Other broad recurrent aberrations included a previously undescribed deletion 2q which appeared to be restricted to patients with t(8;21)-AML (n=5) as well as gains 1q (n=2), 4q (n=3) and 13q (n=3). All patients with gain(13q) had also del(7q) in SNP-array while conventional karyotype showed additional material of unknown origin on the long arm of chromosome 7 [add(7q)]. Whole chromosome 13 painting performed in one of them by fluorescent *in situ* hybridization confirmed transfer of material from chromosome 13 to chromosome 7 leading to both gain(13) and del(7q) (Supplementary Figure 6).

**Table 2:** Recurrent copy number abnormalities in CBF AML patients

Recurrent SNP-array lesions	Start ^¥^	End ^¥^	Size	Gene count	CBF AML	inv(16)	t(8;21)	p-value	
gain(1)(q42.13q44)	227 833 996	249 224 684	21391 kb	197 genes	2 (1%)	0 (0%)	2 (2%)	0.512	
del(2)(q33.2q35)	204 563 014	220 260 561	15698 kb	127 genes	5 (3%)	0 (0%)	5 (4%)	0.078	
del(3)(p13)	71 194 153	71 523 438	329 kb	***FOXP1***	4 (2%)	4 (5%)	0 (0%)	0.028	^*^
gain(4)(q32.1q35.2)	158 379 102	190 957 473	32578 kb	124 genes	3 (2%)	1 (1%)	2 (2%)	1.000	
del(7)(q35q36.1)	147 660 930	151 908 681	4248 kb	71 genes including ***EZH2*** and ***KMT2C***	20 (10%)	10 (12%)	10 (9%)	0.476	
del(8)(q24.11)	117 823 216	117 914 100	91 kb	***RAD21****, RAD21-AS1, MIR3610*	2 (1%)	0 (0%)	2 (2%)	0.512	
focal gain(8)(q24.21)^†^	130 586 319	130 697 500	111 kb	***CCDC26***	9 (5%)	4 (5%)	5 (4%)	1.000	
gain(8)(q24.11q24.3)^†^	118 660 515	140 821 810	22161 kb	92 genes including ***CCDC26*** and ***MYC***	2 (1%)	2 (2%)	0 (0%)	0.170	
trisomy 8^†^	whole chromosome		-	-	14 (7%)	8 (10%)	6 (5%)	0.264	
del(9)(q21.2q21.33)	80 806 493	86 951 615	6145 kb	19 genes including ***TLE1*** and ***TLE4***	14 (7%)	0 (0%)	14 (12%)	<0.001	^*^
trisomy 9	whole chromosome		-	-	2 (1%)	2 (2%)	0 (0%)	0.170	
del(11)(p13)	31 972 741	32 633 735	661 kb	*RCN1,* ***WT1****, WT1-AS, EIF3M, CCDC73*	4 (2%)	2 (2%)	2 (2%)	1.000	
gain(13)(q31.1q34)	85 412 329	115 107 733	29695 kb	135 genes	3 (2%)	1 (1%)	2 (2%)	1.000	
del(17)(q11.2)	29 357 586	29 520 056	162 kb	*MIR4733,* ***NF1***	2 (1%)	2 (2%)	0 (0%)	0.170	
trisomy 21	whole chromosome		-	-	3 (2%)	3 (4%)	0 (0%)	0.070	
trisomy 22	whole chromosome		-	-	11 (6%)	11 (13%)	0 (0%)	<0.001	^*^
del(X)(q26.1)	129 129 272	129 211 954	83 kb	***BCORL1****, ELF4*	2 (1%)	2 (2%)	0 (0%)	0.170	
loss X or Y					63 (32%)	1 (1%)	62 (53%)	<0.001	^*^
loss X^§^	whole chromosome		-	-	22 (24%)	0 (0%)	22 (42%)	<0.001	^*^
loss Y^§^	whole chromosome		-	-	41 (39%)	1 (2%)	40 (63%)	<0.001	^*^
**Breakpoint-associated lesions**									
del(8)(q21.3)	93 096 598	93 128 271	32 kb	***RUNX1T1***	5 (3%)	0 (0%)	5 (4%)	0.078	
del(16)(p13.11)	15 828 494	16 056 322	228 kb	***MYH11,*** *FOPNL, ABCC1*	16 (8%)	16 (20%)	0 (0%)	<0.001	^*^
gain(16)(p13.11)	15 725 039	15 814 747	90 kb	*KIAA0430, NDE1, MIR484,* ***MYH11***	1 (1%)	1 (1%)	0 (0%)	0.414	
del(16)(q22.1)	67 132 654	67 176 123	43 kb	***CBFB****, C16orf70*	13 (7%)	13 (16%)	0 (0%)	<0.001	^*^
gain(16)(q21q22.1)	65 352 347	67 131 638	1779 kb	23 genes including ***CBFB***	1 (1%)	1 (1%)	0 (0%)	0.414	
del(21)(q22.12)	36 183 871	36 210 100	26 kb	***RUNX1***	3 (2%)	0 (0%)	3 (3%)	0.268	
gain(21)(q22.12)	36 355 481	36 423 085	68 kb	***RUNX1,*** *RUNX1-IT1*	2 (1%)	0 (0%)	2 (2%)	0.512	
+der(21) t(8;21)(q22;q22)	whole chromosome		-	*-*	2 (1%)	0 (0%)	2 (2%)	0.512	

* p-value < 0.05.

† Partial chromosomal lesions were considered separately from abnormalities involving the whole chromosome. Gains 8q related to der(21) t(8;21) duplication (n=2) were considered apart from other lesions.

¥ Chromosomal locations were obtained from the Human Feb. 2009 (GRCh37/hg19) assembly of the UCSC Genome Browser (http://genome.ucsc.edu/).

§ Proportions are given within the female population for loss of X and the male population for loss of Y.

### SNP-array identifies recurrent target genes involved in CBF-AML pathogenesis

**Figure 2:**
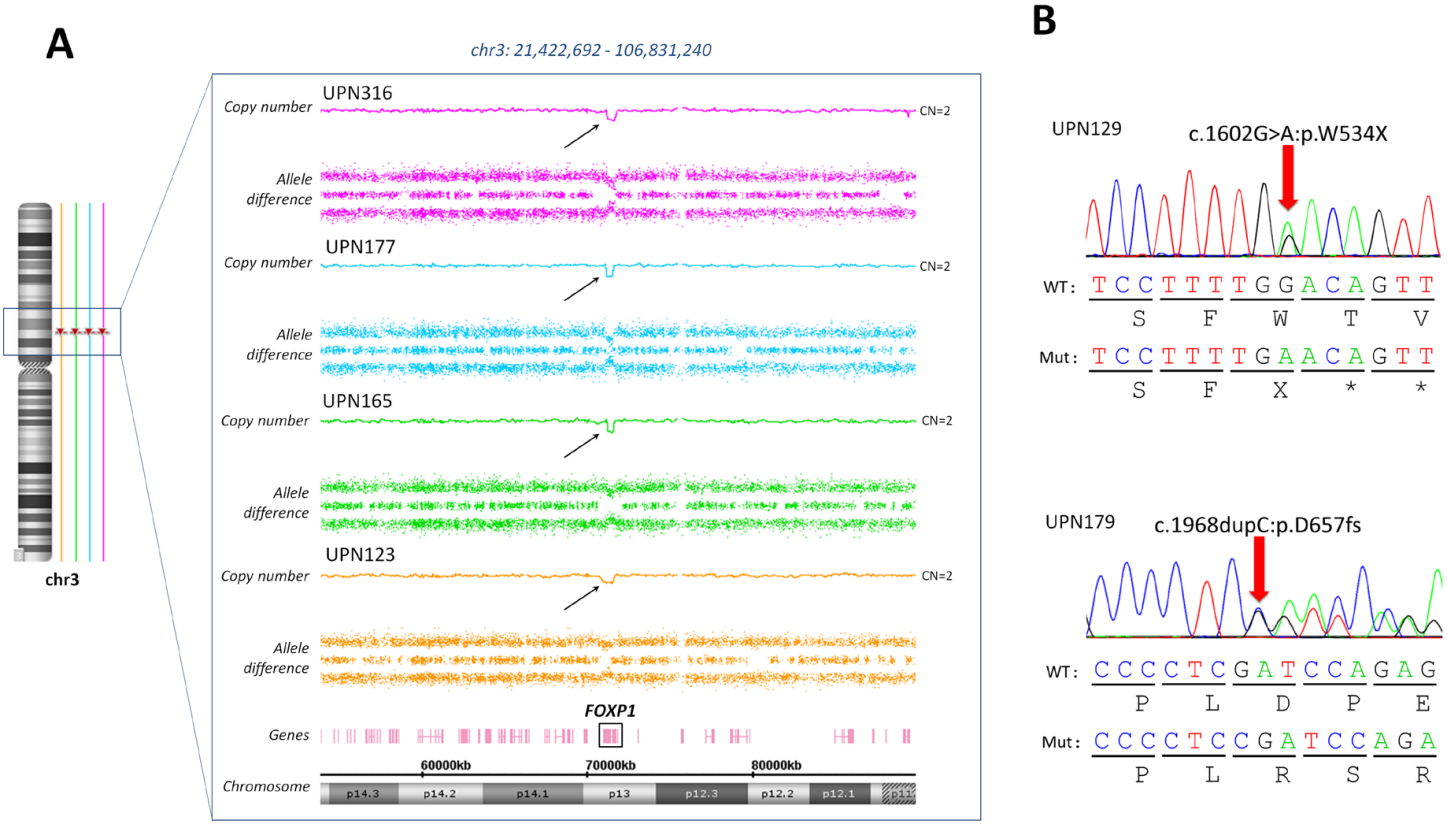
*FOXP1* aberrations in inv(16) AML patients **(A)** Focal deletions targeting the *FOXP1* gene were detected in 4 patients harboring an inv(16). **(B)** Targeted sequencing of *FOXP1* revealed to other inv(16) patients with *FOXP1* truncating mutations.

One of the most common alterations was copy number gains at locus 8q24, which concerned 27 (13.5%) CBF-AML patients through several mechanisms (Supplementary Figure 7): 14 (7%) patients had +8, 4 (2%) patients had broad gains of the long arm of the chromosome 8 and the 9 (4.5%) remaining patients harbored focal gains that contained a single putative gene referred to as *CCDC26* which has been recently linked to myeloid leukemia cell growth [18]. *FOXP1* focal deletions were identified in 4 patients, all with inv(16)-AML. Interestingly, subsequent sequencing of all coding exons of *FOXP1* by high-throughput sequencing (HTS) in the whole cohort identified 2 other inv(16) patients with *FOXP1* truncating mutations (Figure 2). On the other hand, no *FOXP1* deletion or mutation was found in t(8;21) patients.

Other recurrent focal aberrations included deletions of *WT1* (n=4), *BCORL1* (n=2), the cohesin core component *RAD21* (n=2) and the RAS pathway modulator *NF1* (n=2) whose mutations are recurrent in CBF-AML [6, 7]. Additionally, some focal unique CNAs involved highly relevant genes such as deletions of the transcription factors *IKZF1* (n=1) and *ETV6* (n=1), gain of *MYB* (n=1), deletions of the cohesin regulator *PDS5A* (n=1) or the potential tumor suppressor *MGA* (n=1). A gain of *CNOT4*, which amplification is expected to enhance JAK/STAT signaling [19], was seen in one patient as the sole secondary abnormality.

### ZBTB7A is a target of CN-LOH in t(8;21)-AML

**Figure 3:**
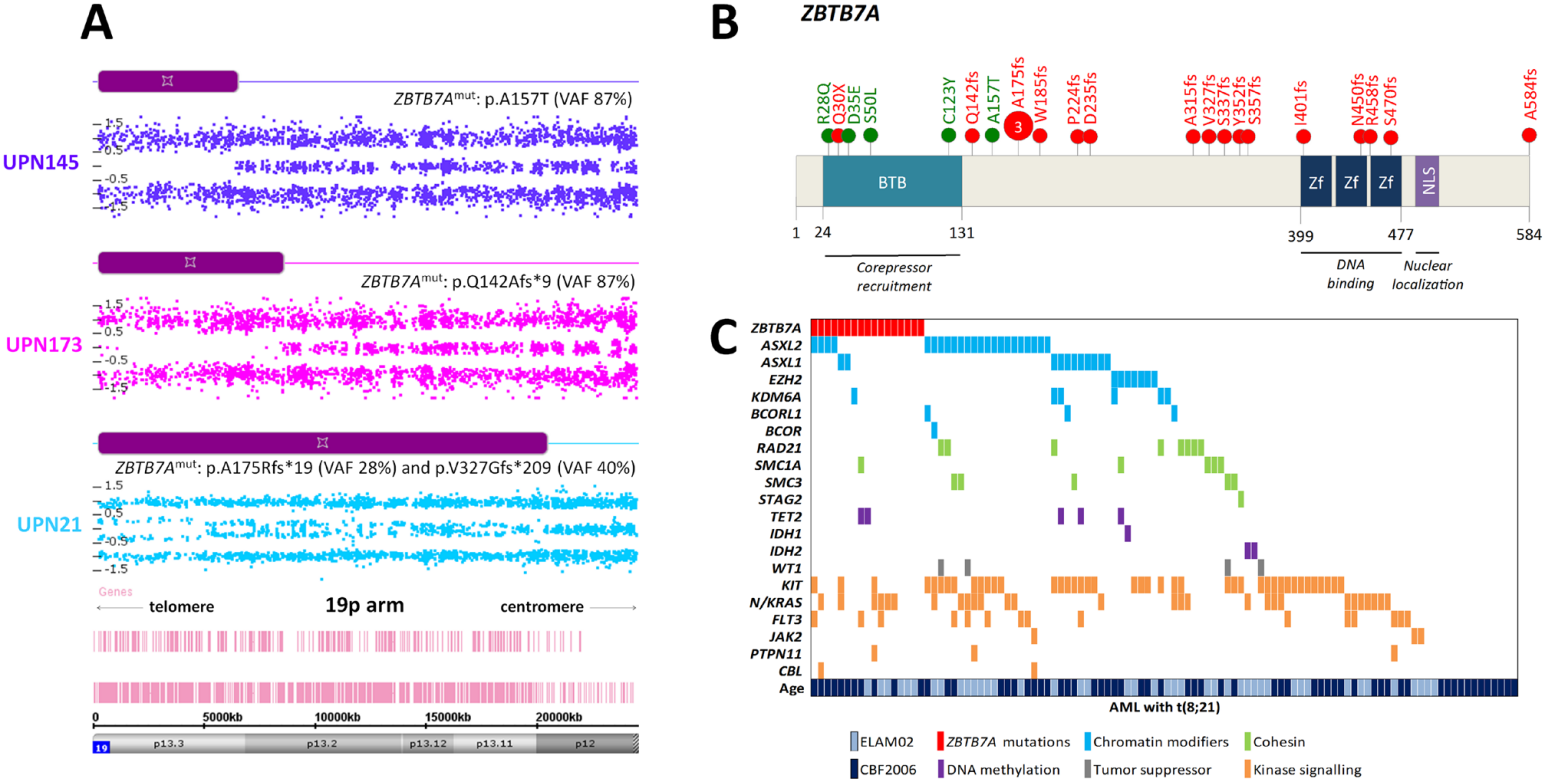
*ZBTB7A* aberrations in AML patients with t(8;21) **(A)** Concomitant mutation and CN-LOH in 3 patients with t(8;21)-AML. **(B)** ZBTB7A protein (NP_056982.1) and identified mutations (red = truncating; green = missense). BTB: BR-C ttk and bab; NLS: nuclear localization sequence; Zf: zinc finger. **(C)** Genomic landscape of t(8;21)-AML including *ZBTB7A* mutations.

The short arm of chromosome 19 was recurrently affected by CN-LOH in 3 patients with restriction to the t(8;21) subtype. The minimal affected region was about 6 Mb and contained 209 genes. Notably, this region was previously reported by Kühn et *al.* in 2 t(8;21)-AML patients with paired samples [17]. This region contained the *ZBTB7A* gene recently described as highly mutated in patients with t(8;21)-AML but not in patients with inv(16)-AML [20, 21]. In order to validate *ZBTB7A* as a target of CN-LOH in 19p, we performed HTS of all coding exons of *ZBTB7A* in the whole cohort. We identified 23 *ZBTB7A* mutations in 19 patients (4 patients had 2 mutations) restricted to the t(8;21) subgroup (16%; Supplementary Table 3). Three patients harbored the same frameshift mutation at alanine 175. All patients with 19p CN-LOH harbored concomitant *ZBTB7A* mutation validating *ZBTB7A* as the target of this aberration (Figure 3A). Missense mutations clustered in the N-terminal BTB domain while frameshift mutations occurred through the whole protein as previously described by others [20, 21] (Figure 3B). We did not identify any association or exclusion with other known mutations (Figure 3C). There was no difference in age, sex or white blood cell count according the *ZBTB7A* mutational status. Finally, there was no impact of *ZBTB7A* mutations on overall survival (OS) and relapse-free survival (RFS) in t(8;21)-AML patients (Supplementary Figure 8).

### CCDC26 disruption is likely to be associated with aberrant tyrosine kinase signaling in CBF-AML

**Figure 4:**
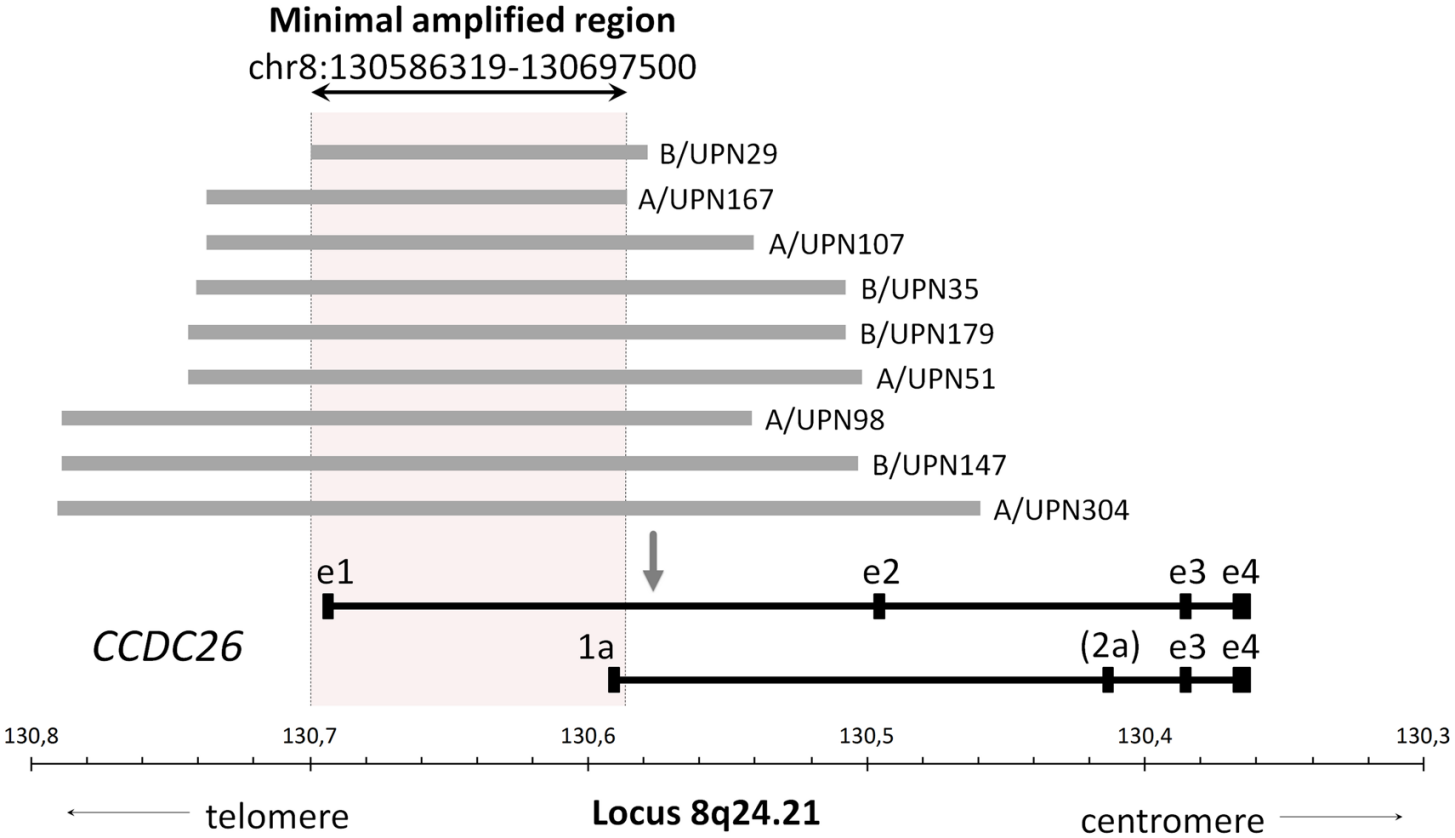
Focal *CCDC26* amplifications Horizontal grey lines illustrate focal recurrent amplifications detected in 9 CBF AML genomes across the *CCDC26* locus. The area inside the dotted lines represents the minimal amplified region shared by all patients. Major variants of CCDC26 mRNA are shown in black below the figure. Exons are indicated by boxes. The long transcript consists of four exons (from e1 to e4) and the short transcripts comprise 3 or 4 exons (from 1a to e4, more or less exon 2a). The arrow shows the retrovirus insertion site defined by Yin et *al* [37].

**Figure 5:**
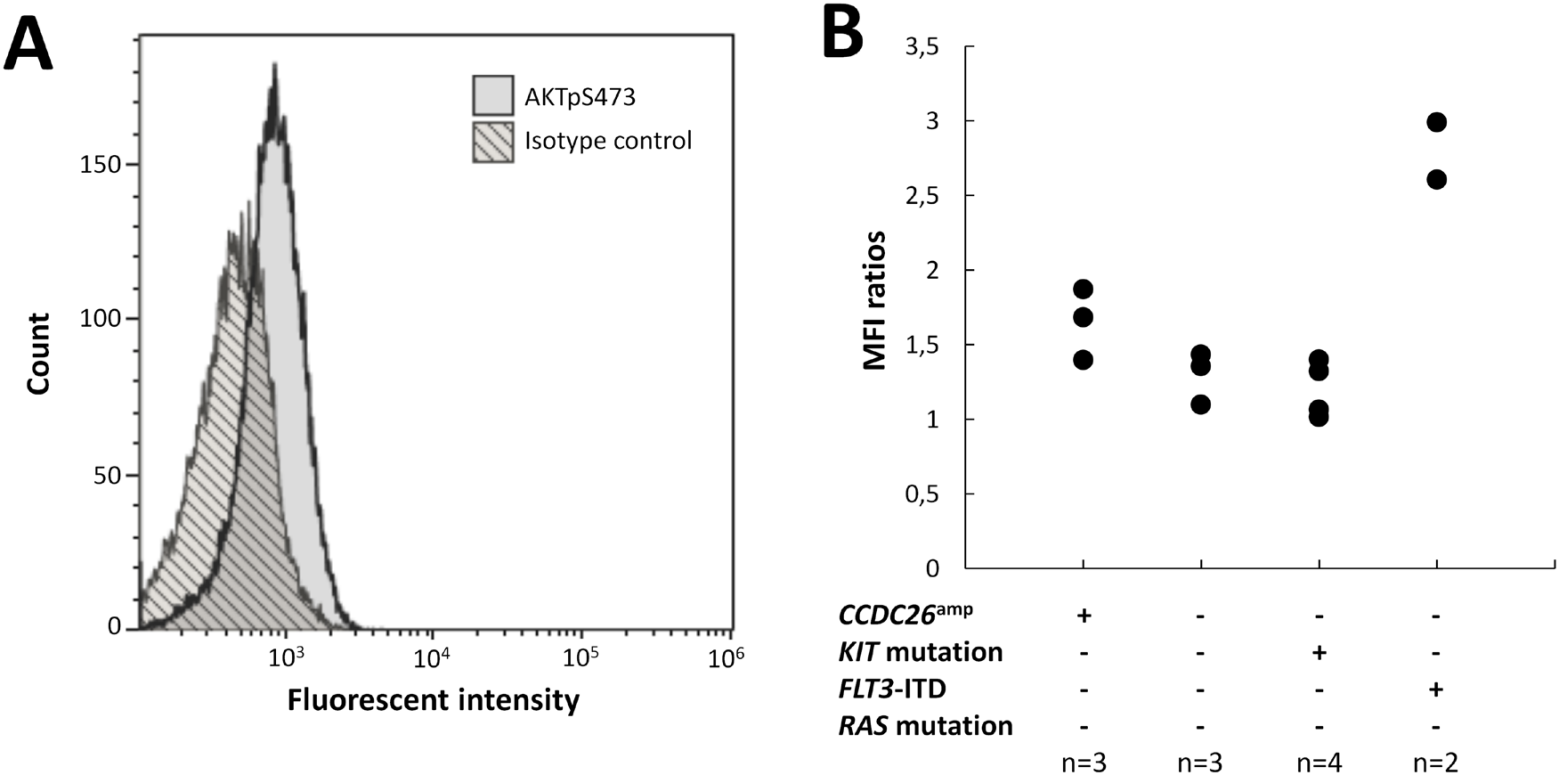
AKTp473 expression in blasts from CBF-AML patients **(A)** AKTpS473 expression level in a patient with *CCDC26* focal amplification (*CCDC26*^amp^). **(B)** AKTpS473 mean fluorescent intensity ratios according to *CCDC26* focal amplification and *KIT/FTL3/RAS* mutational status. Ratios were calculated as (MFI of blast cells)/(MFI of isotype IgG control).

Nine (4.5%) patients harbored focal gains confined to the *CCDC26* locus (Figure 4). Interestingly, it has been recently suggested that *CCDC26* could control myeloid leukemia cell growth through regulation of *KIT* expression [18]. Considering that hyperactive *KIT* mutations are highly prevalent in CBF-AML (about 35% of cases)[6] and that *CCDC26* focal amplification (*CCDC26*^amp^) is found more frequently in CBF-AML than in non CBF-AML [22, 23], these findings could reveal a new lesion associated with aberrant tyrosine kinase pathway activation in CBF-AML patients. Importantly, all but one patient harboring *CCDC26*^amp^ were *KIT* wild-type. In order to explore this hypothesis, expressions of the *KIT* receptor and the phosphorylated downstream effector AKT (AKTp473) were estimated on diagnostic blast cells by flow cytometry in patients with *CCDC26*^amp^ (n=3). Results were compared with blast cells isolated from patient with normal *CCDC26* copy number (*CCDC26*^nor^) and *KIT* mutation (*KIT*^mut^; n=4), *FLT3*-ITD (n=2) or *KIT*/*FLT3* wild-type (*KIT*^wt^/*FLT3*^wt^; n=3). All patients were *NRAS* and *KRAS* wild-type (*RAS*^wt^). Overall, there was no correlation between *KIT* expression and *CCDC26* copy number or *KIT*/*FLT3* mutational status. Median expression of AKTp473 showed a trend of higher expression in *CCDC26*^amp^-cells (+24%) compared with *CCDC26*^nor^/KIT^wt^/FLT3^wt^/RAS^wt^-cells (Figure 5). Although we could not directly linked AKTp473 and *CCDC26*^amp^, these data suggest an underlying mechanism leading to the activation of tyrosine kinase pathway in cells harboring *CCDC26*^amp^. On the other hand, median expression of AKTp473 clearly increased in *CCDC26*^nor^/*KIT*^wt^/*FLT3*-ITD/RAS^wt^-cells (+107%) compared with *CCDC26*^nor^/KIT^wt^/FLT3^wt^/RAS^wt^-cells while it was not observed for *CCDC26*^nor^/KIT^mut^/FLT3^wt^/RAS^wt^-cells.

## DISCUSSION

SNP-array karyotyping of 198 patients with CBF-AML highlight great differences between t(8;21)-AML and inv(16)-AML as described in most recent studies focused on cooperating mutations [6, 7, 20, 21, 24–26]. Notably, these studies identified frequent mutations in genes encoding epigenetic regulators and cohesin complex in t(8;21)-AML while they were absent in inv(16)-AML [6, 7, 20, 21]. Overall, we found more CNAs in t(8;21)-AML than in inv(16)-AML, mostly due to genomic deletions. Excluding breakpoint-associated lesions, the most common CNAs included large chromosomal lesions usually seen by conventional cytogenetics including LOS, del(9q), del(7q) and +8 in t(8;21)-AML compared with +22, del(7q), +8, +21 and +9 in inv(16)-AML. While it is clear that these events are nonrandom and contribute to the pathogenesis of CBF-AML, there was no association between clinical outcome and the number of SNP-array lesions nor the presence of these specific aberrations. Remarkably, there was no significant difference in the number of lesions between adult and pediatric patients suggesting they reflect the same entity and could be studied together in further biological experiments.

Concerning recurrent broad deletions, SNP-array led us to identify MDRs on chromosome 9 (involving *TLE1* and *TLE4*) and chromosome 7 (containing *EZH2* and *KMT2C*). In previous experiments, Dayyani et *al* shown that haploinsufficiency of *TLE1* and *TLE4* could overcome the negative survival and anti-proliferative effects of *RUNX1-RUNXT1* on myeloid progenitors and promote leukemogenesis [13]. Using SNP-array profiling, Kühn et *al* previously identified a MDR on 7q containing only 4 genes including *KMT2C* [17]. By sequence analysis of 46 CBF-AML without *KMT2C* deletion, they identified a single somatic heterozygous frameshift mutation in this gene. More recently, it was shown that *KMT2C* act as a tumor suppressor gene in AML [16]. Together, these data suggest that *KMT2C* haploinsufficiency is likely to be a cooperating event in CBF-AML pathogenesis. While the MDR defined by Kühn et *al* did not contain *EZH2*, by contrast with the present study, the high frequency of polycomb mutations *(ASXL1, ASXL2* and *EZH2)* in t(8;21)-AML suggest that *EZH2* haploinsufficiency could be of interested, at least in t(8;21)-AML patients [6, 27].

Although the number of CNAs was low, our analysis identified recurrent deletions and subsequent mutations in known and potentially new cancer genes. These included deletions in *WT1*, *BCORL1*, *RAD21*, *EZH2* or *NF1* whose mutations have been recurrently found in CBF-AML patients [6, 7, 20]. Interestingly, we identified *FOXP1* aberrations (deletions or truncated mutations) in 7% of patients with inv(16)-AML, arguing for a pathogenic role in this particular subtype. *FOXP1* (*forkhead box P1*) encodes one of the 4 members of the FOXP subfamily of forkhead transcription factors, known to be involved in human malignancies, cell survival and differentiation [28]. *FOXP1* has been described as a target of chromosomal translocations and amplifications in B-cell lymphomas and prostate cancer [28]. By contrast, *FOXP1* losses have been described in clear cell-type kidney cancer but also rarely in myeloproliferative neoplasms [29] and AML with normal [30] or complex karyotype [31]. *FOXP1* has been shown to function as a transcriptional repressor in monocytic differentiation [32]. Thus, it is likely that *FOXP1* loss-of-function could contribute to leukemogenesis especially in inv(16)-AML which is most often diagnosed as AML with a monocytic compartment [1].

Three patients with t(8;21)-AML had CN-LOH of 19p leading to homozygous *ZBTB7A* mutation. *ZBTB7A* (also known as *LRF* or *Pokemon*) encodes a transcription factor of the POK (*poxvirus and zinc finger and Krüppel*)/ZBTB (*zinc finger and broad complex, tramtrack, and bric-a-brac*) family involved in the hematopoietic development and the negative regulation of glycolysis [21]. Sequence analysis of *ZBTB7A* in the whole cohort identified mutations in 16% of t(8;21)-AML while no mutation was found in inv(16)-AML. Our results are in line with other studies previously reporting *ZBTB7A* mutations in 10% to 23% of t(8;21)-AML [7, 20, 21]. Somatic *ZBTB7A* mutations are also reported at low frequencies in various solid malignancies [33]. Missense mutations identified in our analysis clustered in the BTB domain which mediates the homodimerization and/or heterodimerization with other proteins [33]. Truncated mutations were distributed through the whole gene leading to the loss of the zinc-finger domain involved in DNA binding and/or nuclear localization signal. Previous experiments from Hartmann et *al* suggest that *ZBTB7A* act as a tumor suppressor in t(8;21)-AML [20]. Overexpression of *ZBTB7A* in Kasumi-1 cells (cell line harboring a t(8;21) rearrangement) leads to reduced proliferation while its haploinsufficiency should result in the induction of glycolysis promoting tumor progression [20, 33].

Finally, we identified *CCDC26* (*coiled-coil domain containing* 26) focal amplifications in 4.5% of the total cohort, consistent with previous SNP-array investigations showing such lesions in 4.7% of CBF-AML genomes [17, 22]. The nature of *CCDC26* remains ambiguous but it is more plausible that the *CCDC26* locus encodes a long non-coding-RNA [34] involved in tumors, including low-grade gliomas [35] and pancreatic cancer [36]. This locus, also known as *RAM* (*retinoic acid modulator*), was initially reported as required for retinoic acid (RA)-induced myeloid differentiation. Retroviral DNA integration into this locus has been shown to generated RA-resistant cells [37]. Interestingly, Hirano et *al* showed that *CCDC26*-knockdown resulted in *KIT* up-regulation and enhanced survival in myeloid leukemia cell lines. First of all, these results appeared conflicting with our data showing *CCDC26* amplification in CBF-AML. However, this paradox could be explicable by the fact that *CCDC26* amplification does not extend to the whole gene. Partial amplification restricted to exons 1 and 1a could result in *CCDC26* disruption leading to an abnormal mRNA structure without any activity or able to interfere with the remaining intact gene [38]. Thus, considering the high frequency class I mutations (especially in *KIT*, *FLT3* and *RAS* genes) in CBF-AML [6], it is likely that *CCDC26* disruption could highlight a new class I aberration leading to increased cell survival and proliferation in leukemia. In order to explore this hypothesis, we studied phosphoAKT expression by flow cytometry in CBF-AML cells harboring *CCDC26* focal amplification. Although we were able to study only 3 patients with this lesion, blast cells from patients with *CCDC26* disruption showed a subtle increased expression of phosphoAKT compared with blast cells from patients with normal *CCDC26* copy number and no class I mutation (*KIT*, *FLT3* and *RAS* wild-type). However, this was not observed blast cells from 4 *KIT*-mutated patients suggesting other activated pathways associated with *KIT* mutations. By contrast phosphoAKT expression were clearly increased in blast cells from 2 patients with *FLT3-ITD*. Unfortunately, we were not able to study other cytoplasmic effectors such as ERK, SRC or STAT proteins that could be deregulated in leukemia. Also, because we studied a very small number of patients, we were not able to give strong conclusions but our data suggest a mechanism leading to tyrosine kinase signaling in cells with *CCDC26* disruption. Of course, further studies are needed to directly link *CCDC26* disruption and aberrant tyrosine kinase signaling in CBF-AML.

In conclusion, we defined the landscape of SNP-array lesions in a cohort of 198 adult and pediatric CBF-AML at time of diagnosis. As no cell culture is required, we described the frequency of known cytogenetic abnormalities with an unbiased approach and found no association with clinical outcome. Although, the number of SNP-array lesions appeared very low in CBF-AML, when combining with sequence analyses, we were able to identify recurrent involvement of known and potentially new cancer genes including *FOXP1* loss-of-function in inv(16)-AML, *ZBTB7A* homozygous mutations through CN-LOH in t(8;21)-AML and *CCDC26* disruption in both genetic subgroups of CBF-AML. Because of the low frequency of recurrent events, further studies focused on specific genetic subgroups of AML are needed to specify the incidence and the role of these aberrations in leukemogenesis.

## MATERIALS AND METHODS

### Patients and samples

This study focused on diagnostic bone marrow (BM) samples from 198 CBF-AML patients including 116 AML with t(8;21) and 82 AML with inv(16). The cohort included 125 adults (aged from 18 to 60 years) and 73 children (aged from 1 to 17 years) enrolled in the French trials CBF2006 (ClinicalTrials.gov NCT00428558)[5] and ELAM02 (ClinicalTrials.gov NCT00149162) respectively. Median age was 30 years (range: 1-60). Patient’s characteristics are summarized in Supplementary Table 4. Studies were approved by the Ethics Committee of Nîmes University Hospital and by the Institutional Review Board of the French Regulatory Agency and were conducted in accordance with the Declaration of Helsinki. Details about treatment regimens are provided in Supplementary Figure 9 and Supplementary Figure 10.

### SNP-array karyotyping

DNA was extracted from diagnostic cell pellets using the QIAamp Tissue Kit (Qiagen) according to the manufacturer’s instructions. Patient’s genomic DNA was processed and hybridized to Cytoscan HD array (Affymetrix) according to the manufacturer’s protocol. Data were analyzed using the Chromosome Analysis Suite (ChAS) software (Affymetrix). In a first step, only copy number variants with a size over 20 kb including at least 20 consecutive markers as well as CN-LOH over 3 Mb were considered for this analysis. In a second step, we adopted a stringent and conservative algorithm in order to distinguish somatic from constitutional SNP-array lesions. Variations were excluded as known copy number variants if there was more than 50% overlap with variants from the public Database of Genomic Variants. Based on previous studies [39–41], only interstitial CN-LOH over 10 Mb and CN-LOH extending to telomeres were considered to be acquired abnormalities. Remaining CN-LOH were considered as possibly constitutional and then rejected for subsequent analyses. Finally, all CNAs and CN-LOH fulfilling the above criteria were validated by visual inspection and annotated for size, position and location of genes based on the human genome version 19 (hg19) of the UCSC Genome Browser.

### Mutational analysis

Considering data from SNP-array karyotyping, target sequencing was performed for all coding exons of *FOXP1* (NM_001244810) and *ZBTB7A* (NM_015898). Libraries were prepared using the Ampliseq system according to the manufacturer’s instruction and run on Personal Genome Machine (PGM, Life Technologies). Raw HTS data from PGM sequencing were processed by Torrent Browser (Life Technologies) and SeqNext (JSI Medical System). The depth of coverage was more than 2000X for both genes. Variants were confirmed by direct Sanger Sequencing as previously described [27]. Frameshift and nonsense variants were always considered as relevant mutations. Single nucleotide variants were retained in the absence of description into public databases of human polymorphisms and effects on protein function were predicted with SIFT and Polyphen-2.

### Multiparameter flow cytometry

Diagnostic blast cells were obtained from thawed cryopreserved BM samples after red blood cell lysis. Fixation, permeabilization and staining (with both intracellular and cell surface markers) were performed using the PerFix-no centrifuge assay kit (Beckman Coulter) according to the manufacturer’s instructions. The antibody panel contained: anti-AKTpS473-Vio515 (clone: REA359, Miltenyi Biotec), anti-CD33-PC5.5 (clone D3HL60.251, Beckman Coulter), anti-CD34-PC7 (clone 581, Beckman Coulter), anti-CD117/KIT-APC (clone 104D2D1, Beckman Coulter), anti-CD3-AA750 (clone UCHT1, Beckman Coulter), anti-CD4-PB (clone 13B8.2, Beckman Coulter) and anti-CD45-KO (clone J33, Beckman Coulter). Blast cells were gated as CD45^dim^, SSC^low^, CD33+, excluding lymphocytes (CD45^bright^, SSC^low^, CD33−), monocytes (CD45int/bright, SSC^int^, CD33^bright^) and mature myelomonocytic cells (CD45^int^, SSC^high^, CD33^dim/neg^). Isotype control (clone REA293, Miltenyi Biotec) was used to better define the threshold of AKTpS473-positive cells. AKTpS473 expression levels were calculated as [mean fluorescent intensity (MFI) of blast cells/MFI of isotype IgG control]. Measurements were performed on a Navios flow cytometer and analyzed with Kaluza software (Beckman-Coulter).

### Statistical methods

Failure time data were analyzed and compared after censoring at transplant for patients who received allogeneic stem cell transplantation in first complete remission (CR). RFS and OS were estimated by the Kaplan-Meier method. RFS was estimated taking into account death in first CR for competing risk. Comparisons between patient subgroups were performed by the Mann-Whitney test for continuous variables and by Fisher’s exact test for categorical variables. All statistical tests were performed with the SPSS Statistics software (IBM).

## SUPPLEMENTARY MATERIALS FIGURES AND TABLES





## Abbreviations

AML: acute myeloid leukemia
BM: bone marrow
CBF: core binding factor
CNAs: copy-number abnormalities
CN-LOH: copy-neutral losses of heterozygosity
CR: complete remission
HTS: high-throughput sequencing
LOS: loss of a sex chromosome
MFI: mean fluorescent intensity
OS: overall survival
RA: retinoic acid
RFS: relapse-free survival
SNP: single nucleotide polymorphism

## References

[1] Duployez N, Willekens C, Marceau-Renaut A, Boudry-Labis E, Preudhomme C (2015). Prognosis and monitoring of core-binding factor acute myeloid leukemia: current and emerging factors. Expert Rev Hematol.

[2] Downing JR (2003). The core-binding factor leukemias: lessons learned from murine models. Curr Opin Genet Dev.

[3] Wiemels JL, Xiao Z, Buffler PA, Maia AT, Ma X, Dicks BM, Smith MT, Zhang L, Feusner J, Wiencke J, Pritchard-Jones K, Kempski H, Greaves M (2002). In utero origin of t(8;21) AML1-ETO translocations in childhood acute myeloid leukemia. Blood.

[4] Miyamoto T, Nagafuji K, Akashi K, Harada M, Kyo T, Akashi T, Takenaka K, Mizuno S, Gondo H, Okamura T, Dohy H, Niho Y (1996). Persistence of multipotent progenitors expressing AML1/ETO transcripts in long-term remission patients with t(8;21) acute myelogenous leukemia. Blood.

[5] Jourdan E, Boissel N, Chevret S, Delabesse E, Renneville A, Cornillet P, Blanchet O, Cayuela JM, Recher C, Raffoux E, Delaunay J, Pigneux A, Bulabois CE (2013). Prospective evaluation of gene mutations and minimal residual disease in patients with core binding factor acute myeloid leukemia. Blood.

[6] Duployez N, Marceau-Renaut A, Boissel N, Petit A, Bucci M, Geffroy S, Lapillonne H, Renneville A, Ragu C, Figeac M, Celli-Lebras K, Lacombe C, Micol JB (2016). Comprehensive mutational profiling of core binding factor acute myeloid leukemia. Blood.

[7] Faber ZJ, Chen X, Gedman AL, Boggs K, Cheng J, Ma J, Radtke I, Chao JR, Walsh MP, Song G, Andersson AK, Dang J, Dong L (2016). The genomic landscape of core-binding factor acute myeloid leukemias. Nat Genet.

[8] Schlenk RF, Benner A, Krauter J, Büchner T, Sauerland C, Ehninger G, Schaich M, Mohr B, Niederwieser D, Krahl R, Pasold R, Döhner K, Ganser A (2004). Individual patient data-based meta-analysis of patients aged 16 to 60 years with core binding factor acute myeloid leukemia: a survey of the German Acute Myeloid Leukemia Intergroup. J Clin Oncol.

[9] Marcucci G, Mrózek K, Ruppert AS, Maharry K, Kolitz JE, Moore JO, Mayer RJ, Pettenati MJ, Powell BL, Edwards CG, Sterling LJ, Vardiman JW, Schiffer CA (2005). Prognostic factors and outcome of core binding factor acute myeloid leukemia patients with t(8;21) differ from those of patients with inv(16): a Cancer and Leukemia Group B study. J Clin Oncol.

[10] Appelbaum FR, Kopecky KJ, Tallman MS, Slovak ML, Gundacker HM, Kim HT, Dewald GW, Kantarjian HM, Pierce SR, Estey EH (2006). The clinical spectrum of adult acute myeloid leukaemia associated with core binding factor translocations. Br J Haematol.

[11] Cazier JB, Holmes CC, Broxholme J (2012). GREVE: Genomic Recurrent Event ViEwer to assist the identification of patterns across individual cancer samples. Bioinformatics.

[12] Sweetser DA, Peniket AJ, Haaland C, Blomberg AA, Zhang Y, Zaidi ST, Dayyani F, Zhao Z, Heerema NA, Boultwood J, Dewald GW, Paietta E, Slovak ML (2005). Delineation of the minimal commonly deleted segment and identification of candidate tumor-suppressor genes in del(9q) acute myeloid leukemia. Genes Chromosomes Cancer.

[13] Dayyani F, Wang J, Yeh JR, Ahn EY, Tobey E, Zhang DE, Bernstein ID, Peterson RT, Sweetser DA (2008). Loss of TLE1 and TLE4 from the del(9q) commonly deleted region in AML cooperates with AML1-ETO to affect myeloid cell proliferation and survival. Blood.

[14] Mikulasovich M, LeBlanc A, Scalise A, Manwani D, Keyzner A, Najfeld V (2009). Duplication and triplication of der(21) t(8;21)(q22;q22) in acute myeloid leukemia. Cancer Genet Cytogenet.

[15] Wang X, Dai H, Wang Q, Wang Q, Xu Y, Wang Y, Sun A, Ruan J, Chen S, Wu D (2013). EZH2 mutations are related to low blast percentage in bone marrow and -7/del(7q) in de novo acute myeloid leukemia. PLoS One.

[16] Chen C, Liu Y, Rappaport AR, Kitzing T, Schultz N, Zhao Z, Shroff AS, Dickins RA, Vakoc CR, Bradner JE, Stock W, LeBeau MM, Shannon KM (2014). MLL3 is a haploinsufficient 7q tumor suppressor in acute myeloid leukemia. Cancer Cell.

[17] Kühn MW, Radtke I, Bullinger L, Goorha S, Cheng J, Edelmann J, Gohlke J, Su X, Paschka P, Pounds S, Krauter J, Ganser A, Quessar A (2012). High-resolution genomic profiling of adult and pediatric core-binding factor acute myeloid leukemia reveals new recurrent genomic alterations. Blood.

[18] Hirano T, Yoshikawa R, Harada H, Harada Y, Ishida A, Yamazaki T (2015). Long noncoding RNA, CCDC26, controls myeloid leukemia cell growth through regulation of KIT expression. Mol Cancer.

[19] Grönholm J, Kaustio M, Myllymäki H, Kallio J, Saarikettu J, Kronhamn J, Valanne S, Silvennoinen O, Rämet M (2012). Not4 enhances JAK/STAT pathway-dependent gene expression in Drosophila and in human cells. FASEB J.

[20] Hartmann L, Dutta S, Opatz S, Vosberg S, Reiter K, Leubolt G, Metzeler KH, Herold T, Bamopoulos SA, Bräundl K, Zellmeier E, Ksienzyk B, Konstandin NP (2016). ZBTB7A mutations in acute myeloid leukaemia with t(8;21) translocation. Nat Commun.

[21] Lavallée VP, Lemieux S, Boucher G, Gendron P, Boivin I, Armstrong RN, Sauvageau G, Hébert J (2016). RNA-sequencing analysis of core binding factor AML identifies recurrent ZBTB7A mutations and defines RUNX1-CBFA2T3 fusion signature. Blood.

[22] Radtke I, Mullighan CG, Ishii M, Su X, Cheng J, Ma J, Ganti R, Cai Z, Goorha S, Pounds SB, Cao X, Obert C, Armstrong J (2009). Genomic analysis reveals few genetic alterations in pediatric acute myeloid leukemia. Proc Natl Acad Sci U S A.

[23] Renneville A, Abdelali RB, Chevret S, Nibourel O, Cheok M, Pautas C, Dulery R, Boyer T, Cayuela JM, Hayette S, Raffoux E, Farhat H, Boissel N (2013). Clinical impact of gene mutations and lesions detected by SNP-array karyotyping in acute myeloid leukemia patients in the context of gemtuzumab ozogamicin treatment: results of the ALFA-0701 trial. Oncotarget.

[24] Eisfeld AK, Kohlschmidt J, Schwind S, Nicolet D, Blachly JS, Orwick S, Shah C, Bainazar M, Kroll KW, Walker CJ, Carroll AJ, Powell BL, Stone RM (2017). Mutations in the CCND1 and CCND2 genes are frequent events in adult patients with t(8;21)(q22;q22) acute myeloid leukemia. Leukemia.

[25] Krauth MT, Eder C, Alpermann T, Bacher U, Nadarajah N, Kern W, Haferlach C, Haferlach T, Schnittger S (2014). High number of additional genetic lesions in acute myeloid leukemia with t(8;21)/RUNX1-RUNX1T1: frequency and impact on clinical outcome. Leukemia.

[26] Paschka P, Du J, Schlenk RF, Gaidzik VI, Bullinger L, Corbacioglu A, Späth D, Kayser S, Schlegelberger B, Krauter J, Ganser A, Köhne CH, Held G (2013). Secondary genetic lesions in acute myeloid leukemia with inv(16) or t(16;16): a study of the German-Austrian AML Study Group (AMLSG). Blood.

[27] Micol JB, Duployez N, Boissel N, Petit A, Geffroy S, Nibourel O, Lacombe C, Lapillonne H, Etancelin P, Figeac M, Renneville A, Castaigne S, Leverger G (2014). Frequent ASXL2 mutations in acute myeloid leukemia patients with t(8;21)/RUNX1-RUNX1T1 chromosomal translocations. Blood.

[28] Katoh M, Igarashi M, Fukuda H, Nakagama H, Katoh M (2013). Cancer genetics and genomics of human FOX family genes. Cancer Lett.

[29] Klampfl T, Harutyunyan A, Berg T, Gisslinger B, Schalling M, Bagienski K, Olcaydu D, Passamonti F, Rumi E, Pietra D, Jäger R, Pieri L, Guglielmelli P (2011). Genome integrity of myeloproliferative neoplasms in chronic phase and during disease progression. Blood.

[30] Bullinger L, Krönke J, Gaidzik V, Döhner H, Döhner K (2010). Comment on ‘integrative genomic profiling of human prostate cancer.’ Leukemia.

[31] Milosevic JD, Puda A, Malcovati L, Berg T, Hofbauer M, Stukalov A, Klampfl T, Harutyunyan AS, Gisslinger H, Gisslinger B, Burjanivova T, Rumi E, Pietra D (2012). Clinical significance of genetic aberrations in secondary acute myeloid leukemia. Am J Hematol.

[32] Shi C, Sakuma M, Mooroka T, Liscoe A, Gao H, Croce KJ, Sharma A, Kaplan D, Greaves DR, Wang Y, Simon DI (2008). Down-regulation of the forkhead transcription factor Foxp1 is required for monocyte differentiation and macrophage function. Blood.

[33] Liu XS, Liu Z, Gerarduzzi C, Choi DE, Ganapathy S, Pandolfi PP, Yuan ZM (2016). Somatic human ZBTB7A zinc finger mutations promote cancer progression. Oncogene.

[34] Hirano T, Siregar Y (2013). Is CCDC26 a Novel Cancer-Associated Long-Chain Non-Coding RNA?. Oncogene and Cancer - From Bench to Clinic.

[35] Shete S, Hosking FJ, Robertson LB, Dobbins SE, Sanson M, Malmer B, Simon M, Marie Y, Boisselier B, Delattre JY, Hoang-Xuan K, Hallani SE, Idbaih A (2009). Genome-wide association study identifies five susceptibility loci for glioma. Nat Genet.

[36] Peng W, Jiang A (2016). Long noncoding RNA CCDC26 as a potential predictor biomarker contributes to tumorigenesis in pancreatic cancer. Biomed Pharmacother.

[37] Yin W, Rossin A, Clifford JL, Gronemeyer H (2006). Co-resistance to retinoic acid and TRAIL by insertion mutagenesis into RAM. Oncogene.

[38] Hirano T (2015). The role of the CCDC26 long noncoding RNA as a tumor suppressor. RNA Dis.

[39] Tiu RV, Gondek LP, O’Keefe CL, Huh J, Sekeres MA, Elson P, McDevitt MA, Wang XF, Levis MJ, Karp JE, Advani AS, Maciejewski JP (2009). New lesions detected by single nucleotide polymorphism array–based chromosomal analysis have important clinical impact in acute myeloid leukemia. J Clin Oncol.

[40] O’Keefe C, McDevitt MA, Maciejewski JP (2010). Copy neutral loss of heterozygosity: a novel chromosomal lesion in myeloid malignancies. Blood.

[41] Heinrichs S, Li C, Look AT (2010). SNP array analysis in hematologic malignancies: avoiding false discoveries. Blood.

