# Supplementary material for: SNP-array lesions in core binding factor acute myeloid leukemia
Source: Oncotarget. 2018 Jan 8;9(5):6478–89. doi: 10.18632/oncotarget.24031 (PMC5814226; doi:10.18632/oncotarget.24031)
Supplement: Supplementary file 1 [file oncotarget-09-6478-s001.pdf]

SNP-array lesions in core binding factor acute myeloid leukemia

REFERENCES

1. Duployez N, Marceau-Renaut A, Boissel N, Petit A, Bucci M, Geffroy S, Lapillonne H, Renneville A, Ragu C, Figeac M, Celli-Lebras K, Lacombe C, Micol JB, et al. Comprehensive mutational profiling of core binding factor acute myeloid leukemia. Blood. 2016; 127: 2451–9. https://doi.org/10.1182/blood-2015-12-688705.

2. Cazier JB, Holmes CC, Broxholme J. GREVE: Genomic Recurrent Event ViEwer to assist the identification of patterns across individual cancer samples. Bioinformatics. 2012; 28: 2981–2.

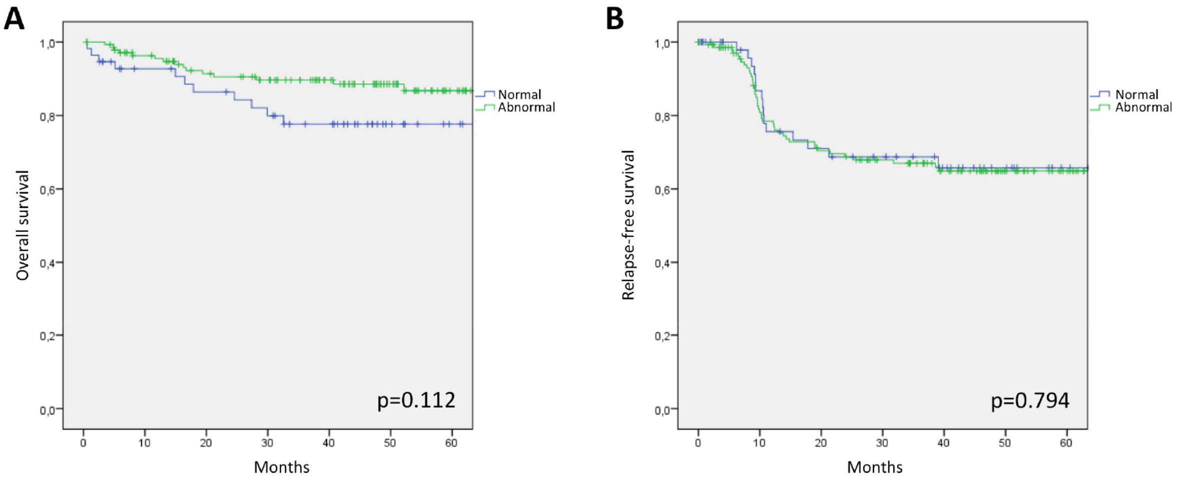

Supplementary Figure 1: Clinical outcome according to SNP-a profile. Overall survival **(A)** and relapse-free survival **(B)** in CBF AML according to the presence (abnormal) or absence (normal) of SNP-array lesions. Patients who received allogeneic stem cell transplantation were censored at time of transplant.

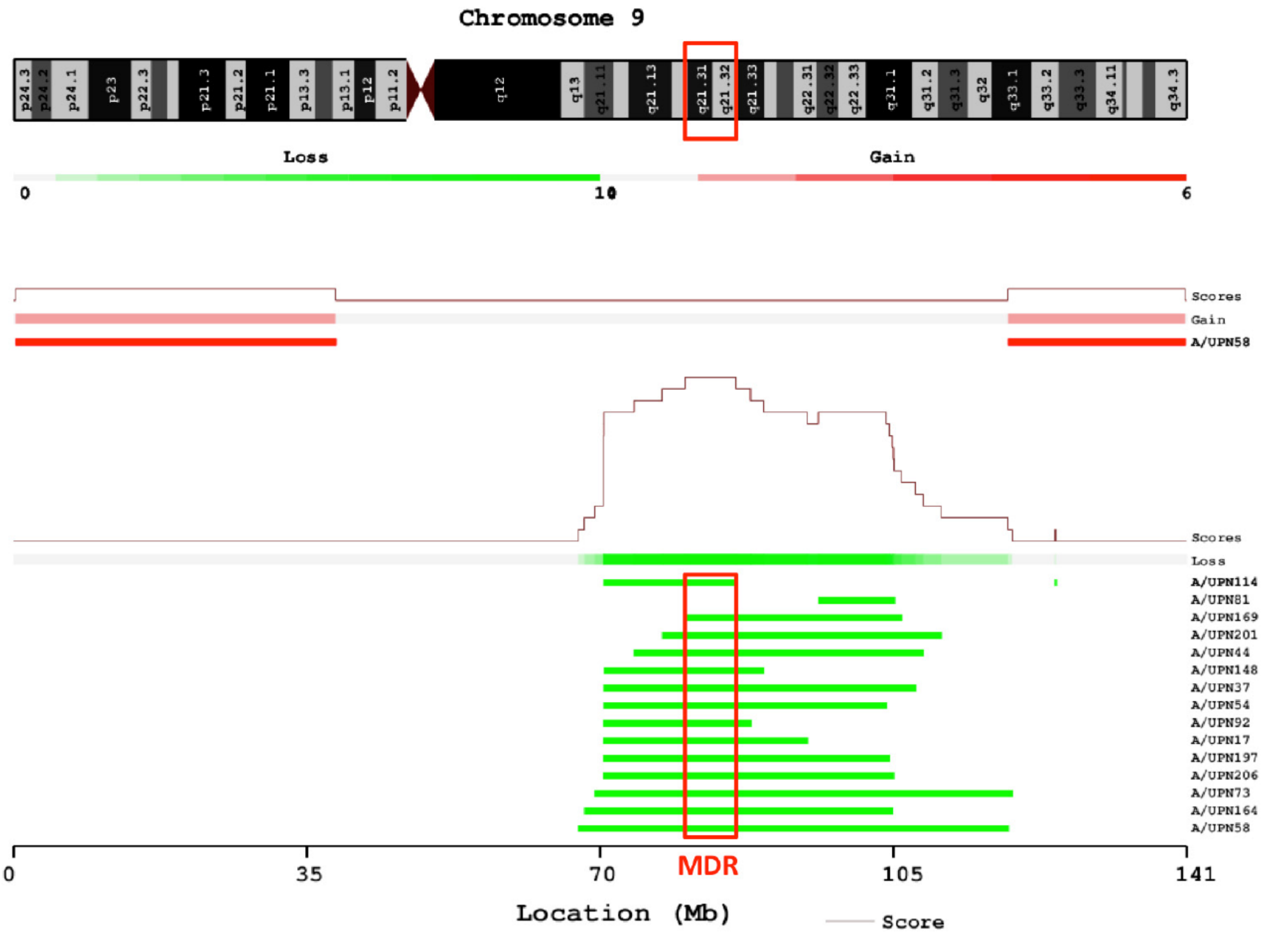

Supplementary Figure 2: Deletion 9q. Interstitial deletion of the long arm of chromosome 9 occurred in 15 cases, all of which were t(8;21) AML (referred as "A" next to UPNs). The minimal deleted region (MDR, showing the highest score), shared by 14 of 15 patients, was of 6.1 Mb in size (location: chr9:80 806 493-86 951 615) and contained 19 genes (including *TLE1* and *TLE4*). A last patient (UPN81) had a more distal del(9)(q22.32q31.1) of 9.1 Mb in size containing 84 genes. UPN58 had probable duplication of the der(9)del(9)(q13q33.1) showing gains of the non-deleted regions. (Figure restricted to patients with del(9q) designed with the GREVE application [2]: http://www.well.ox.ac.uk/GREVE).

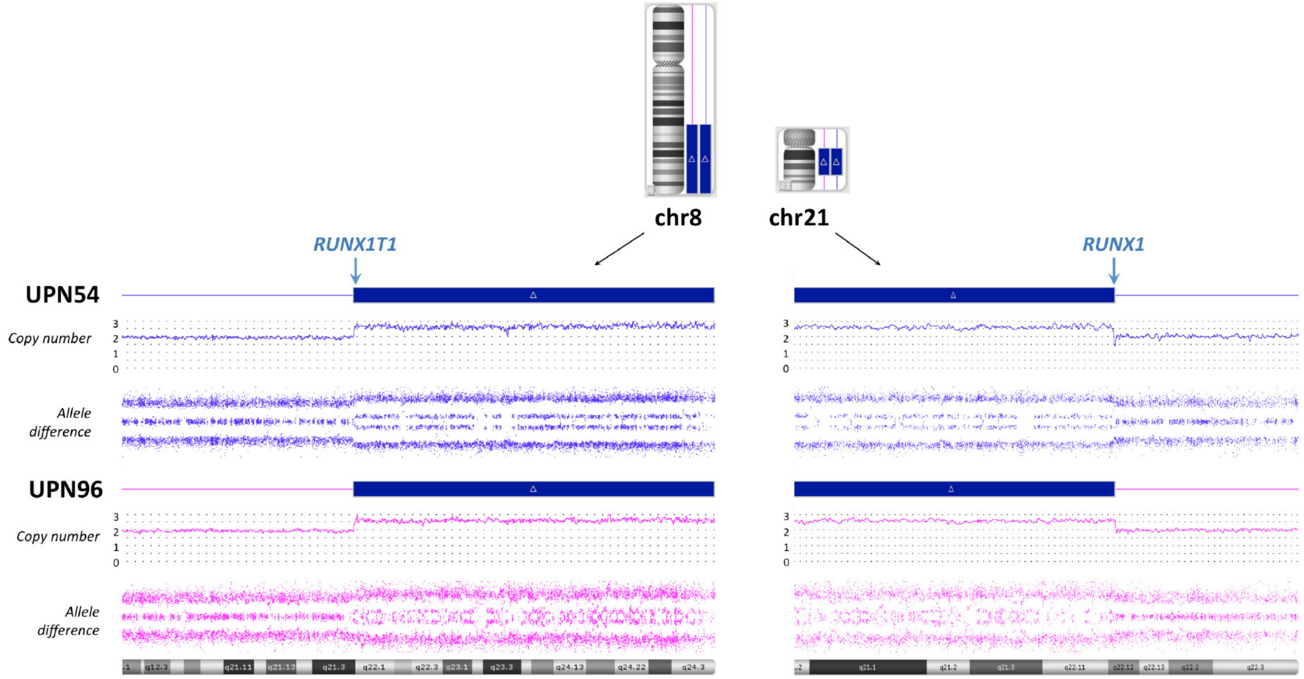

Supplementary Figure 3: Duplication of the der(21)t(8;21). One pediatric patient (UPN54) and one adult patient (UPN96) showed trisomy of the regions upstream of the *RUNX1* gene and downstream of the *RUNX1T1* gene. Conventional karyotype confirmed the duplication of the der(21)t(8;21) in both patients as the sole additional abnormality in UPN96 and associated with del(9q) and loss of Y in UPN54.

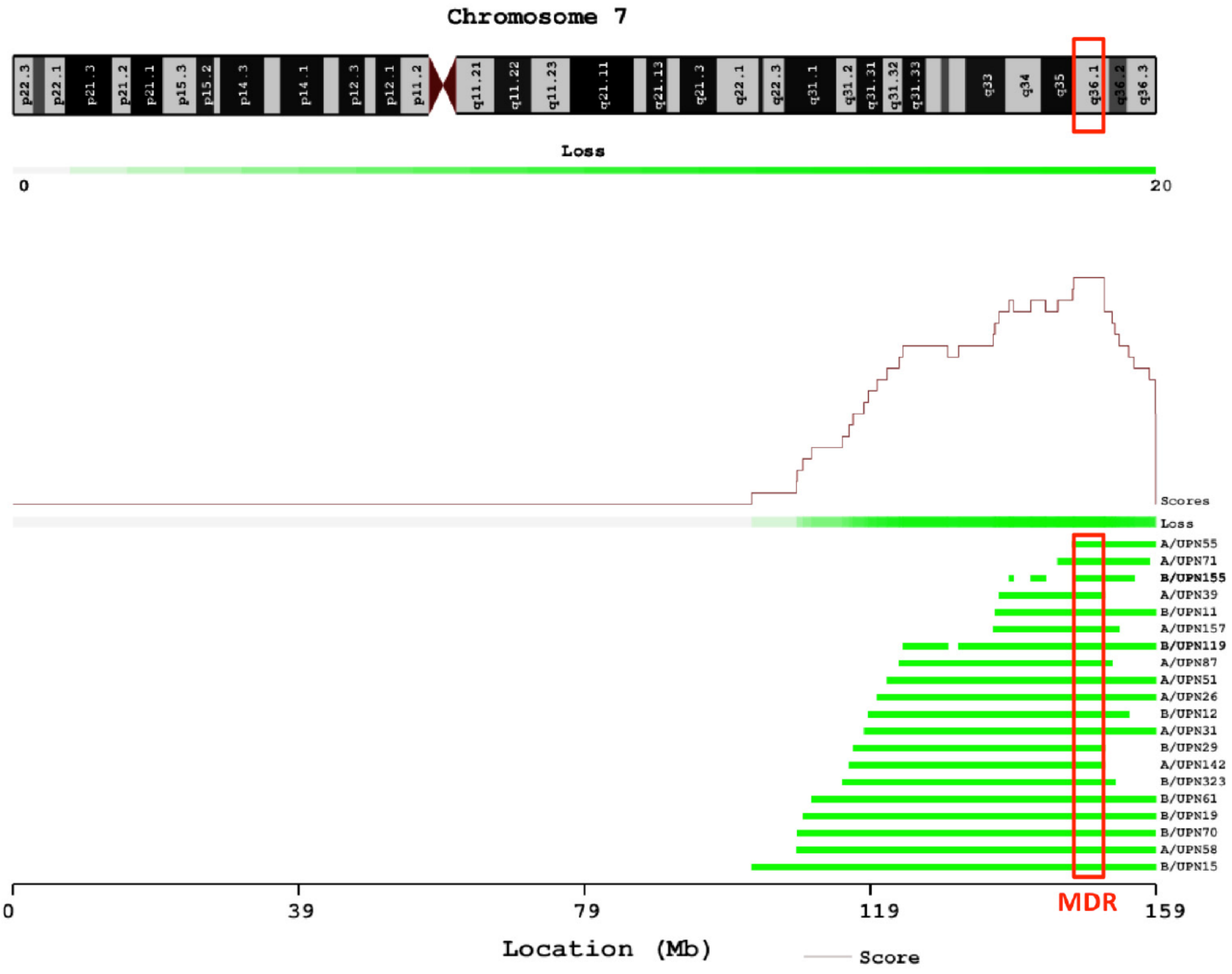

Supplementary Figure 4: Deletion 7q. Interstitial deletion of the long arm of chromosome 7 occurred in 20 cases, including 10 cases with t(8;21) and 10 cases with inv(16) (referred as "A" and "B" next to UPNs). The MDR (showing the highest score), shared by all patients, was of 4.2 Mb in size (location: chr7:147 660 930-151 908 681) and contained 71 genes (including *EZH2* and *KMT2C*). (Figure restricted to patients with del(7q) designed with the GREVE application [2]: http://www.well.ox.ac.uk/GREVE).

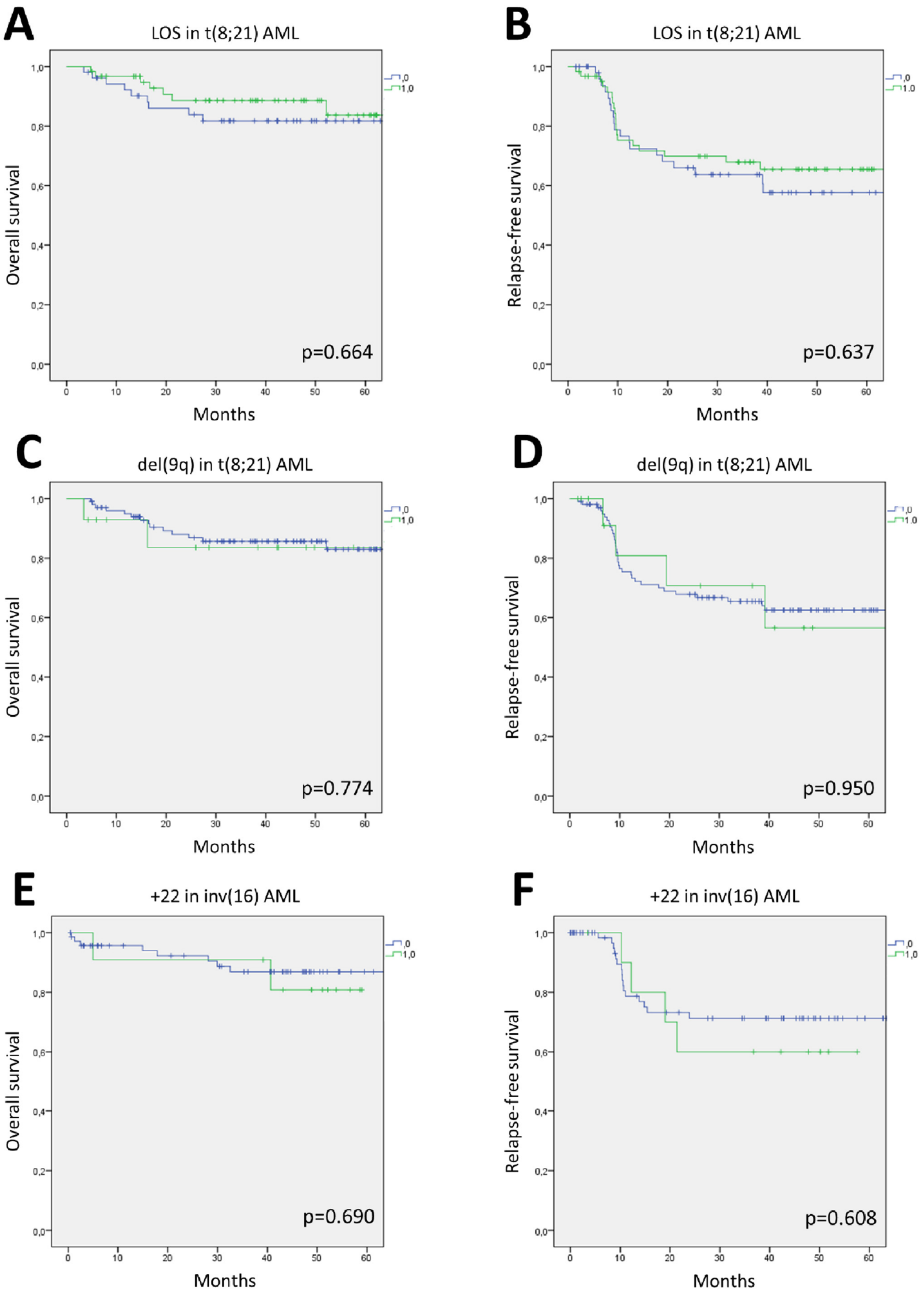
A
LOS in t(8;21) AML
Overall survival
Months
p=0.664
B
LOS in t(8;21) AML
Relapse-free survival
Months
p=0.637
C
del(9q) in t(8;21) AML
Overall survival
Months
p=0.774
D
del(9q) in t(8;21) AML
Relapse-free survival
Months
p=0.950
E
+22 in inv(16) AML
Overall survival
Months
p=0.690
F
+22 in inv(16) AML
Relapse-free survival
Months
p=0.608

(*Continued*)

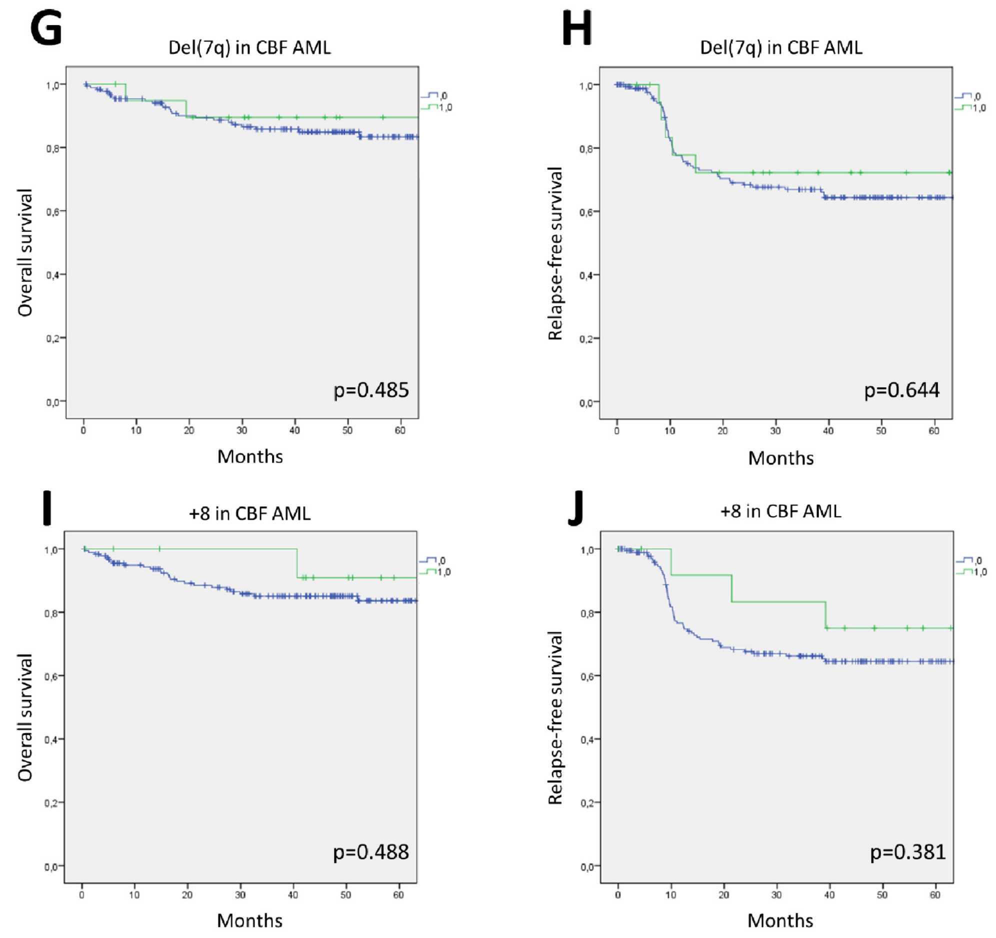

Supplementary Figure 5: Clinical outcome according to recurrent genetic aberrations. Recurrent genetic aberrations observed in at least 10 patients were studied for their impact on outcome: loss of sex chromosome **(A, B)**, del(9q) **(C, D)**, trisomy 22 **(E, F)**, del(7q) **(G, H)** and trisomy 8 **(I, J)**. Patients who received allogeneic stem cell transplantation were censored at time of transplant. Green curves refer to patients with the aberration.

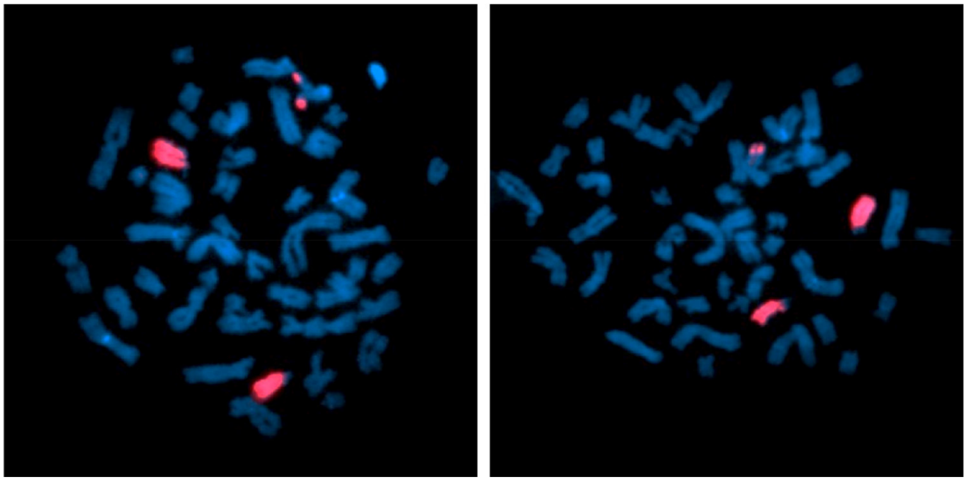

Supplementary Figure 6: Whole chromosome 13 painting in a patient with gain(13q) and del(7q). Whole chromosome 13 painting (Metasystems) by fluorescent *in situ* hybridization was applied to fixated metaphases cells according to standard procedures. The result showed transfer of material from chromosome 13 to chromosome 7.

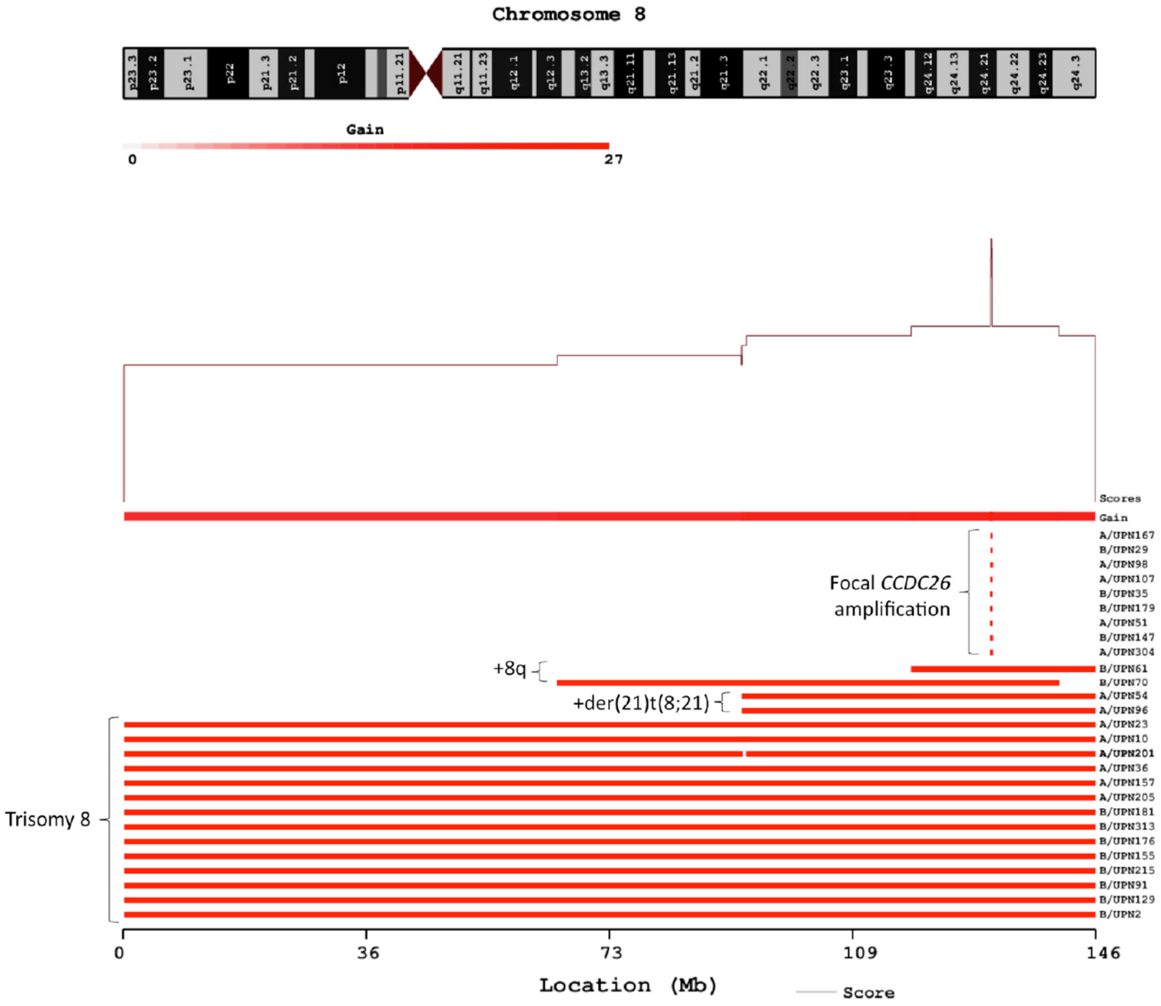

Supplementary Figure 7: Gain 8q. Amplification of the 8q24 was found in 27 cases, including 13 cases with t(8;21) and 14 cases with inv(16) (referred as “A” and “B” next to UPNs). Fourteen cases had trisomy 8. Two inv(16) AML patients had broad gains of the long arm of chromosome 8 (8q). Two t(8;21) AML patients had gains 8q from the *RUNX1T1* gene to the telomere region, related to the duplication of the der(21)t(8;21). Finally, focal gains focused on CCDC26 concerned 9 cases. (Figure restricted to patients with gains 8q designed with the GREVE application [2]: http://www.well.ox.ac.uk/GREVE).

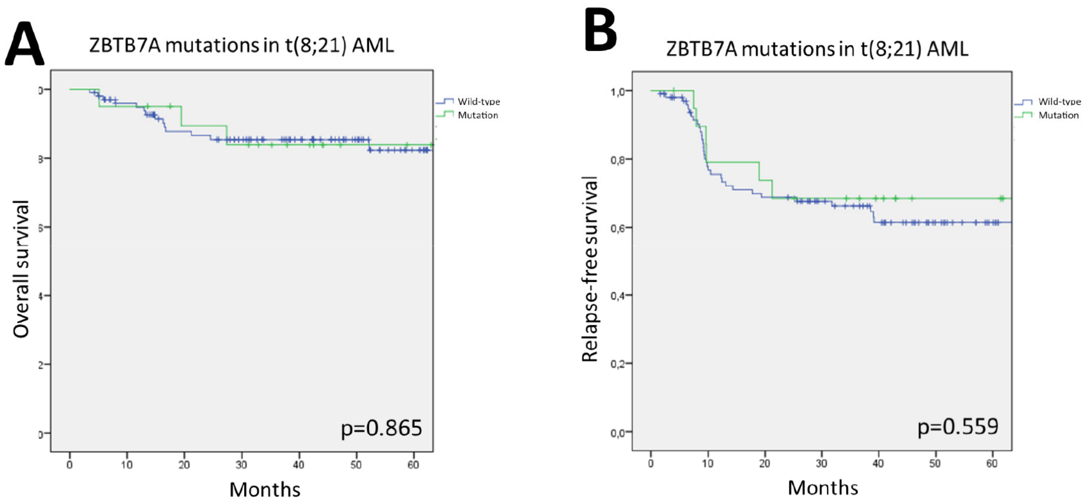

Supplementary Figure 8: Clinical outcome according to *ZBTB7A* mutations in patients with t(8;21). (A) Overall survival. **(B)** Relpase-free survival.

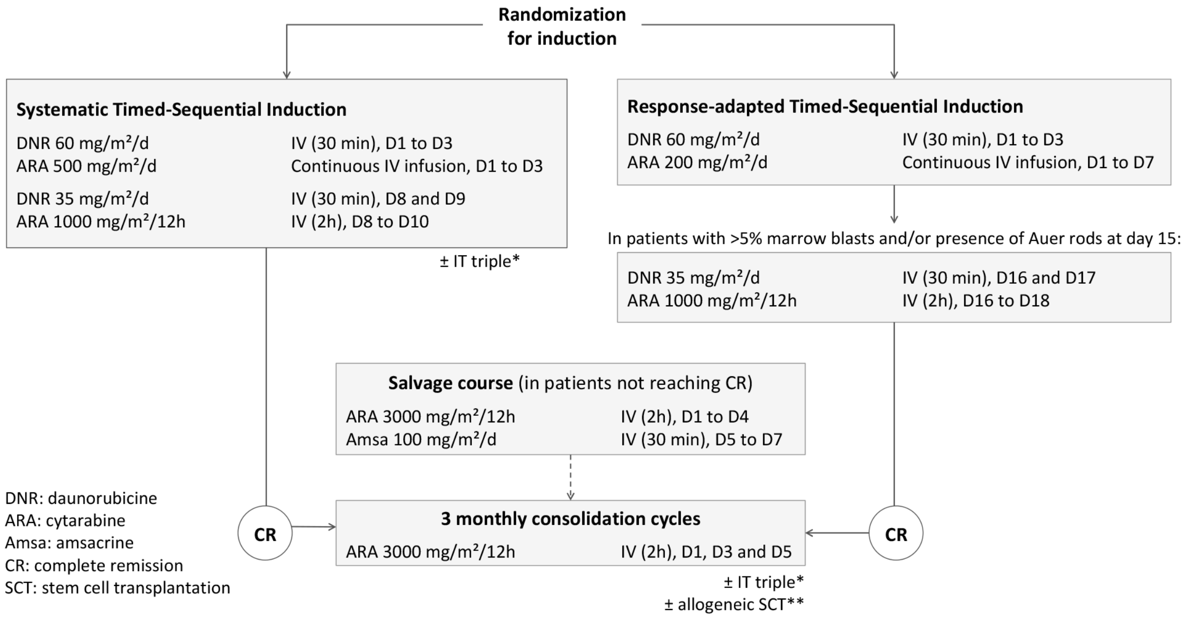

*Patients with CBFB-MYH11-AML and WBC > 100.10^9/L received CNS prophylaxis with 4 triple intrathecal infusions (methotrexate 15 mg, cytarabine 40 mg, methylprednisolone 40 mg).

** Patients not reaching at least a 3-log reduction in minimal residual disease ratio before initiation of the 2nd consolidation cycle were candidates for allogeneic SCT in first CR if they had a matched sibling or 10/10 HLA allele fully matched unrelated donor, as were those who needed the salvage course to reach CR.

Supplementary Figure 9: CBF2006 trial design.

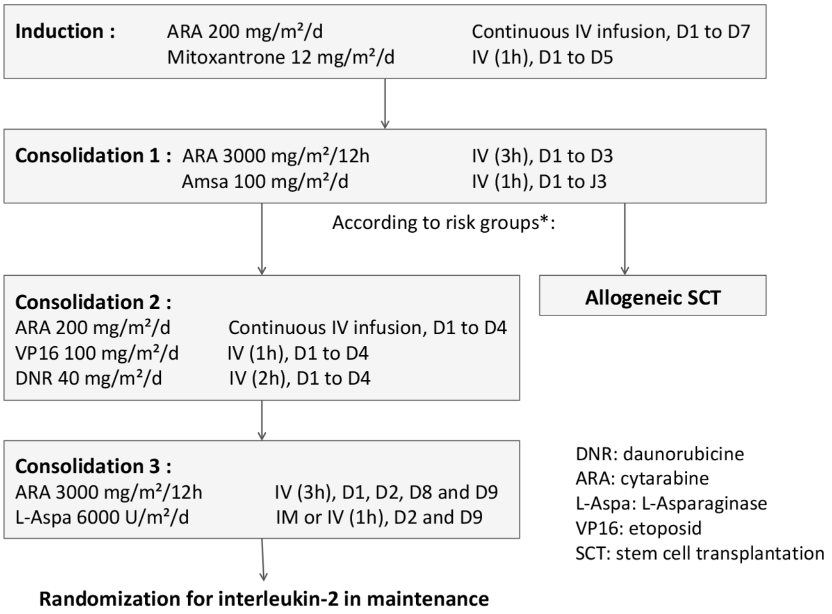

* Allogeneic SCT was not recommended in first CR in CBF AML patients reaching at least a 3-log reduction in minimal residual disease ratio at the end of the first consolidation.

Supplementary Figure 10: ELAM02 trial design.

Supplementary Table 1: Patient characteristics according to CBF AML subtype

	CBF-AML	AML with inv(16)	AML with t(8;21)	p-value	
**Patients, n**	198	82	116		
**Median age, y [range]**	30 [1–60]	33 [1–60]	28 [2–60]	0.554	
**Median WBC, x10^9/L [range]**	16,8 [1, 3–215]	38,7 [1, 9–215]	12,8 [1, 3–163]	<0.001	*
**Gender (male/female)**	105/93	42/40	63/53	0.773	
**Trial (CBF2006/ELAM02)**	125/73	52/30	73/43	1.000	
**Outcome**					
Deaths, n (%)	27 (14)	10 (12)	17 (15)	0.679	
Relapses, n (%)	59 (30)	20 (24)	39 (34)	0.207	
**Gene mutations**					
KIT, n (%)	68/176 (39)	28/75 (37)	40/101 (40)	0.876	
FLT3-TKD, n (%)	21/176 (12)	17/75 (23)	4/101 (4)	<0.001	*
FLT3-ITD, n (%)	12/176 (7)	2/75 (3)	10/101 (10)	0.073	
NRAS, n (%)	50/176 (28)	26/75 (35)	24/101 (24)	0.130	
KRAS, n (%)	24/176 (14)	17/75 (23)	7/101 (7)	0.004	*
ASXL1, n (%)	10/176 (6)	0/75 (0)	10/101 (10)	0.005	*
ASXL2, n (%)	22/176 (13)	0/75 (0)	22/101 (22)	<0.001	*

* p-value < 0.05.

Supplementary Table 2: CBF AML cases studied by SNP-array

See Supplementary File 1

Supplementary Table 3: Mean number of SNP-array lesions per CBF AML case according to trial

	CBF AML	Adults (CBF2006)	Children (ELAM02)	p-value
Patients, n	198	125	73	
Number of CNAs†, mean (range)	1.19 (0-6)	1.18 (0-6)	1.22 (0-5)	0.804
Gains†, mean (range)	0.43 (0-4)	0.42 (0-4)	0.45 (0-3)	0.567
Losses†, mean (range)	0.76 (0-4)	0.75 (0-4)	0.77 (0-3)	0.582
Number of CN-LOH†, mean (range)	0.21 (0-6)	0.19 (0-3)	0.25 (0-6)	0.798
Breakpoint lesions, mean (range)	0.21 (0-2)	0.16 (0-2)	0.29 (0-2)	0.087
Total CNAs/CN-LOH†, mean (range)	1.40 (0-7)	1.37 (0-7)	1.47 (0-7)	0.778

CNA: copy number abnormality; CN-LOH: copy neutral-loss of heterozygosity.
† excluding breakpoint-associated lesions.

Supplementary Table 4: *ZBTB7A* variants (NM_015898) in CBF AML

UPN	CBF	Type	Exon	Nuc. change	AA change	VAF	Polyphen-2	SIFT	CN-LOH
UPN151	t(8;21)	Frameshift	2	c.1067_1068insTGTTC	p.S357Vfs*71	31%	NA	NA	
UPN145	t(8;21)	Single AA change	2	c.469G>A	p.A157T	87%	probably damaging	tolerated	Yes
UPN39	t(8;21)	Frameshift	2	c.551dupC	p.W185Vfs*9	32%	NA	NA	
UPN203	t(8;21)	Frameshift	2	c.522dupC	p.A175Rfs*19	30%	NA	NA	
UPN78	t(8;21)	Frameshift	2	c.1054dupT	p.Y352Lfs*188	55%	NA	NA	
UPN149	t(8;21)	Frameshift	2	c.522dupC	p.A175Rfs*19	33%	NA	NA	
UPN173	t(8;21)	Frameshift	2	c.423dupG	p.Q142Afs*9	87%	NA	NA	Yes
UPN36	t(8;21)	Nonsense	2	c.88C>T	p.Q30X	36%	NA	NA	
UPN171	t(8;21)	Single AA change	2	c.368G>A	p.C123Y	22%	probably damaging	damaging	
UPN301	t(8;21)	Frameshift	2	c.703delG	p.D235Tfs*89	20%	NA	NA	
UPN63	t(8;21)	Single AA change	2	c.149C>T	p.S50L	19%	probably damaging	damaging	
UPN307	t(8;21)	Frameshift	2	c.1200_1201delCA	p.I401Pfs*138	26%	NA	NA	
UPN190	t(8;21)	Frameshift	3	c.1349dupA	p.N450Kfs*90	35%	NA	NA	
UPN192	t(8;21)	Frameshift	2	c.1008_1009insG	p.S337Efs*203	48%	NA	NA	
		Frameshift	3	c.1407dupC	p.S470Qfs*70	24%	NA	NA	
UPN30	t(8;21)	Single AA change	2	c.105C>A	p.D35E	20%	probably damaging	damaging	
		Single AA change	2	c.83G>A	p.R28Q	23%	probably damaging	damaging	
UPN79	t(8;21)	Frameshift	3	c.1372delC	p.R458Afs*99	20%	NA	NA	
		Frameshift	2	c.944_966del	p.A315Dfs*217	26%	NA	NA	
UPN21	t(8;21)	Frameshift	2	c.522dupC	p.A175Rfs*19	28%	NA	NA	Yes
		Frameshift	2	c.980_990del	p.V327Gfs*209	40%	NA	NA	
UPN308	t(8;21)	Frameshift	3	c.1749_1750insTGGGG	p.A584Wfs*62	26%	NA	NA	
UPN1	t(8;21)	Frameshift	2	c.671_676delinsTCGGTGA	p.P224Lfs*26	22%	NA	NA	

AA: amino-acid; CN-LOH: copy neutral-loss of heterozygosity; NA: not applicable; UPN: unit patient number; VAF: variant allele frequency.
