# Supplementary material for: SNP-array lesions in core binding factor acute myeloid leukemia
Source: Oncotarget. 2018 Jan 8;9(5):6478–89. doi: 10.18632/oncotarget.24031 (PMC5814226; doi:10.18632/oncotarget.24031)
Supplement: Supplementary file 2 [file oncotarget-09-6478-s002.doc]

### Supplementary Table 1: CBF AML cases studied by SNP-array.

| **Unit patient number** | **CBF-AML subtype** | **Trial** | **Associated mutations*** | **Type** | **Chr** | **Start** | **End** | **Cytoband Start** | **Cytoband End** | **Gene count** |
| --- | --- | --- | --- | --- | --- | --- | --- | --- | --- | --- |
| UPN2 | inv(16) | ELAM02 | ***KIT*** | Gain | 8 | 158048 | 146295771 | p23.3 | q24.3 | 837 genes |
| UPN4 | inv(16) | ELAM02 | ***NRAS*** | CN-LOH | 4 | 57162562 | 69613721 | q12 | q13.2 | 37 genes |
|  |  |  |  | CN-LOH | 5 | 33405596 | 46383335 | p13.3 | p11 | 77 genes |
|  |  |  |  | CN-LOH | 5 | 49560858 | 94195589 | q11.1 | q15 | 220 genes |
|  |  |  |  | CN-LOH | 19 | 35501050 | 53518514 | q13.12 | q13.41 | 660 genes |
| UPN7 | inv(16) | ELAM02 | ***KIT, WT1*** | CN-LOH | 3 | 74400687 | 111036206 | p12.3 | q13.13 | 108 genes |
| UPN11 | inv(16) | ELAM02 | ***KIT, KRAS*** | Loss | 7 | 136719201 | 159119707 | q33 | q36.3 | 216 genes |
|  |  |  |  | Loss | 16 | 15822262 | 16309046 | p13.11 | p13.11 | *MYH11, FOPNL, ABCC1, ABCC6* |
|  |  |  |  | Loss | 16 | 67121000 | 69058522 | q22.1 | q22.1 | 59 genes |
| UPN12 | inv(16) | ELAM02 | None | Loss | 7 | 119105753 | 155299937 | q31.31 | q36.3 | 311 genes |
|  |  |  |  | Loss | 16 | 15822262 | 16282869 | p13.11 | p13.11 | *MYH11, FOPNL, ABCC1, ABCC6* |
|  |  |  |  | Loss | 16 | 67094752 | 67205134 | q22.1 | q22.1 | *CBFB, C16orf70, B3GNT9, TRADD, FBXL8, HSF4, NOL3* |
|  |  |  |  | Loss | 18 | 56119560 | 56328420 | q21.31 | q21.32 | *ALPK2* |
| UPN15 | inv(16) | ELAM02 | ***NRAS*** | Loss | 7 | 102855853 | 159119707 | q22.1 | q36.3 | 406 genes |
|  |  |  |  | Loss | 16 | 15822262 | 16056322 | p13.11 | p13.11 | *MYH11, FOPNL, ABCC1* |
|  |  |  |  | Loss | 16 | 67129890 | 67327926 | q22.1 | q22.1 | 18 genes |
|  |  |  |  | Gain | X | 153437483 | 155233731 | q28 | q28 | 54 genes |
| UPN19 | inv(16) | ELAM02 | None | Loss | 7 | 109973243 | 159119707 | q31.1 | q36.3 | 364 genes |
|  |  |  |  | Gain | 11 | 98562564 | 134938470 | q22.1 | q25 | 316 genes |
| UPN20 | inv(16) | ELAM02 | ***KIT*** | Loss | X | 129129272 | 129211954 | q26.1 | q26.1 | *BCORL1, ELF4* |
| UPN24 | inv(16) | ELAM02 | ***KRAS*** | Loss | 16 | 15828494 | 16292516 | p13.11 | p13.11 | *MYH11, FOPNL, ABCC1, ABCC6* |
|  |  |  |  | Loss | 16 | 67120714 | 68185160 | q22.1 | q22.1 | 49 genes |
| UPN27 | inv(16) | ELAM02 | ***KIT, NRAS*** | Loss | 17 | 29357586 | 29520056 | q11.2 | q11.2 | *MIR4733, NF1* |
| UPN29 | inv(16) | ELAM02 | None | Loss | 7 | 116975763 | 151956453 | q31.2 | q36.1 | 302 genes |
|  |  |  |  | Gain | 8 | 130577381 | 130697500 | q24.21 | q24.21 | *CCDC26* |
|  |  |  |  | Loss | 16 | 15822262 | 16238533 | p13.11 | p13.11 | *MYH11, FOPNL, ABCC1* |
| UPN35 | inv(16) | ELAM02 | ***KIT, NRAS*** | Gain | 8 | 130505871 | 130742165 | q24.21 | q24.21 | *CCDC26* |
|  |  |  |  | Gain | 22 | 16888899 | 51197838 | q11.1 | q13.33 | 544 genes |
| UPN38 | inv(16) | ELAM02 | ***NRAS*** | Gain | 16 | 65352347 | 67131638 | q21 | q22.1 | 23 genes |
| UPN41 | inv(16) | ELAM02 | ***KRAS*** | Gain | 16 | 15725039 | 15814747 | p13.11 | p13.11 | *KIAA0430, NDE1, MIR484, MYH11* |
| UPN47 | inv(16) | ELAM02 | ***NRAS, FLT3-TKD*** | Gain | 1 | 95954848 | 118715512 | p21.3 | p12 | 184 genes |
| UPN48 | inv(16) | ELAM02 | None | Gain | 22 | 16888899 | 51197838 | q11.1 | q13.33 | 542 genes |
| UPN57 | inv(16) | ELAM02 | ***NRAS*** | Loss | 1 | 48634962 | 48687441 | p33 | p33 | *SKINTL* |
| UPN61 | inv(16) | ELAM02 | ***NRAS*** | Loss | 4 | 917520 | 2773309 | p16.3 | p16.3 | 36 genes |
|  |  |  |  | Loss | 7 | 111146177 | 159119707 | q31.1 | q36.3 | 363 genes |
|  |  |  |  | Gain | 8 | 118660515 | 146295771 | q24.11 | q24.3 | 204 genes |
|  |  |  |  | Gain | 13 | 85412329 | 115107733 | q31.1 | q34 | 135 genes |
|  |  |  |  | Loss | 15 | 38113520 | 63293181 | q14 | q22.2 | 234 genes |
|  |  |  |  | Loss | 16 | 15822262 | 16268230 | p13.11 | p13.11 | *MYH11, FOPNL, ABCC1, ABCC6* |
|  |  |  |  | Loss | 16 | 67132328 | 67770396 | q22.1 | q22.1 | 33 genes |
| UPN62 | inv(16) | ELAM02 | None | Loss | 16 | 15822262 | 16309165 | p13.11 | p13.11 | *MYH11, FOPNL, ABCC1, ABCC6* |
|  |  |  |  | Loss | 16 | 29679183 | 30177807 | p11.2 | p11.2 | 27 genes |
|  |  |  |  | Loss | 16 | 67132654 | 67959737 | q22.1 | q22.1 | 40 genes |
| UPN66 | inv(16) | ELAM02 | ***KIT*** | CN-LOH | 2 | 150328911 | 242775910 | q23.2 | q37.3 | 615 genes |
| UPN70 | inv(16) | ELAM02 | ***KIT, KRAS*** | Loss | 7 | 109183153 | 159065750 | q31.1 | q36.3 | 365 genes |
|  |  |  |  | Gain | 8 | 65333232 | 140821810 | q12.3 | q24.3 | 334 genes |
|  |  |  |  | CN-LOH | 8 | 140699534 | 146292734 | q24.3 | q24.3 | 115 genes |
|  |  |  |  | Loss | 16 | 15822262 | 18172468 | p13.11 | p12.3 | 13 genes |
|  |  |  |  | Loss | 16 | 67132654 | 67310564 | q22.1 | q22.1 | 16 genes |
|  |  |  |  | Loss | 16 | 88133334 | 89140675 | q24.2 | q24.3 | 19 genes |
| UPN91 | inv(16) | CBF2006 | ***KIT, KRAS*** | Gain | 8 | 158048 | 146295771 | p23.3 | q24.3 | 837 genes |
|  |  |  |  | CN-LOH | 12 | 66956987 | 77452223 | q14.3 | q21.2 | 56 genes |
|  |  |  |  | Gain | 21 | 15006457 | 48097372 | q11.2 | q22.3 | 293 genes |
|  |  |  |  | Gain | X | 19560495 | 19951834 | p22.12 | p22.12 | *SH3KBP1, CXorf23* |
| UPN99 | inv(16) | CBF2006 | None | Gain | 22 | 16888899 | 51197838 | q11.1 | q13.33 | 544 genes |
| UPN102 | inv(16) | CBF2006 | ***FLT3-TKD, FLT3-ITD, IDH1*** | Loss | Y | 2650140 | 28799937 | p11.31 | q11.23 | 102 genes |
| UPN103 | inv(16) | CBF2006 | None | Gain | X | 372029 | 155233731 | p22.33 | q28 | 1016 genes |
| UPN111 | inv(16) | CBF2006 | None | Gain | 7 | 134963824 | 135082953 | q33 | q33 | *CNOT4* |
| UPN113 | inv(16) | CBF2006 | None | Loss | 16 | 15822262 | 16282869 | p13.11 | p13.11 | *MYH11, FOPNL, ABCC1, ABCC6* |
|  |  |  |  | Loss | 16 | 67115221 | 67176123 | q22.1 | q22.1 | *CBFB, C16orf70* |
| UPN115 | inv(16) | CBF2006 | ***FLT3-TKD, KDM6A, BCORL1*** | Loss | 16 | 15822262 | 16272796 | p13.11 | p13.11 | *MYH11, FOPNL, ABCC1, ABCC6* |
|  |  |  |  | Loss | 16 | 67131672 | 68503203 | q22.1 | q22.1 | 55 genes |
|  |  |  |  | Loss | X | 129129272 | 129238635 | q26.1 | q26.1 | *BCORL1, ELF4* |
| UPN119 | inv(16) | CBF2006 | ***NRAS*** | Gain | 4 | 158379102 | 190957473 | q32.1 | q35.2 | 124 genes |
|  |  |  |  | Loss | 6 | 96230752 | 127204103 | q16.1 | q22.33 | 142 genes |
|  |  |  |  | CN-LOH | 6 | 126914613 | 170908114 | q22.32 | q27 | 260 genes |
|  |  |  |  | Loss | 7 | 123841390 | 130126000 | q31.33 | q32.2 | 56 genes |
|  |  |  |  | Loss | 7 | 131652054 | 159119707 | q32.3 | q36.3 | 241 genes |
|  |  |  |  | Gain | X | 21933728 | 22092240 | p22.11 | p22.11 | *SMS, PHEX* |
| UPN123 | inv(16) | CBF2006 | ***KRAS, FLT3-TKD*** | CN-LOH | 1 | 882802 | 6317420 | p36.33 | p36.31 | 102 genes |
|  |  |  |  | Loss | 3 | 70886303 | 71594471 | p13 | p13 | *FOXP1, MIR1284* |
| UPN129 | inv(16) | CBF2006 | ***NRAS, KRAS*** | Loss | 2 | 238633686 | 242783384 | q37.3 | q37.3 | 64 genes |
|  |  |  |  | Gain | 8 | 158048 | 146295771 | p23.3 | q24.3 | 837 genes |
|  |  |  |  | Gain | 22 | 16888899 | 51197838 | q11.1 | q13.33 | 537 genes |
| UPN146 | inv(16) | CBF2006 | ***KIT, FLT3-TKD*** | CN-LOH | 2 | 15702 | 70642807 | p25.3 | p13.3 | 418 genes |
|  |  |  |  | Loss | 16 | 15822262 | 16309165 | p13.11 | p13.11 | *MYH11, FOPNL, ABCC1, ABCC6* |
|  |  |  |  | Loss | 16 | 67131672 | 67204111 | q22.1 | q22.1 | *CBFB, C16orf70, B3GNT9, TRADD, FBXL8, HSF4* |
|  |  |  |  | Gain | 22 | 16888899 | 51197838 | q11.1 | q13.33 | 544 genes |
| UPN147 | inv(16) | CBF2006 | None | Gain | 8 | 130501929 | 130789472 | q24.21 | q24.21 | *GSDMC, CCDC26* |
|  |  |  |  | Gain | 9 | 203861 | 141020389 | p24.3 | q34.3 | 961 genes |
|  |  |  |  | Loss | 16 | 15822262 | 16388244 | p13.11 | p13.11 | *MYH11, FOPNL, ABCC1, ABCC6, NOMO3* |
|  |  |  |  | Loss | 16 | 67115389 | 67343493 | q22.1 | q22.1 | 18 genes |
|  |  |  |  | Loss | 17 | 29016359 | 29796197 | q11.2 | q11.2 | 13 genes |
| UPN150 | inv(16) | CBF2006 | ***KIT, NRAS*** | Gain | 22 | 16888899 | 51197838 | q11.1 | q13.33 | 544 genes |
|  |  |  |  | Gain | X | 6446579 | 8135644 | p22.31 | p22.31 | *VCX3A, HDHD1, MIR4767, STS, VCX, PNPLA4, MIR651* |
| UPN155 | inv(16) | CBF2006 | None | Gain | 3 | 114773302 | 115791115 | q13.31 | q13.31 | *ZBTB20, GAP43, LSAMP* |
|  |  |  |  | Loss | 7 | 138705675 | 139279250 | q34 | q34 | 11 genes |
|  |  |  |  | Loss | 7 | 141738012 | 143778966 | q34 | q35 | 40 genes |
|  |  |  |  | Loss | 7 | 147660930 | 156085669 | q35 | q36.3 | 87 genes |
|  |  |  |  | Gain | 8 | 158048 | 146295771 | p23.3 | q24.3 | 837 genes |
|  |  |  |  | Loss | 11 | 31972741 | 32633735 | p13 | p13 | *RCN1, WT1, WT1-AS, EIF3M, CCDC73* |
|  |  |  |  | CN-LOH | 19 | 28273327 | 59097752 | q11 | q13.43 | 974 genes |
| UPN160 | inv(16) | CBF2006 | ***KIT, NRAS, FLT3-TKD*** | Gain | 6 | 163598835 | 163820968 | q26 | q26 | *PACRG, PACRG-AS1, DKFZp451B082* |
|  |  |  |  | Gain | 9 | 203861 | 141020389 | p24.3 | q34.3 | 961 genes |
|  |  |  |  | Loss | 16 | 15822262 | 16309046 | p13.11 | p13.11 | *MYH11, FOPNL, ABCC1, ABCC6* |
|  |  |  |  | Loss | 16 | 67131672 | 67506080 | q22.1 | q22.1 | 23 genes |
|  |  |  |  | Gain | 22 | 16888899 | 51197838 | q11.1 | q13.33 | 544 genes |
| UPN162 | inv(16) | CBF2006 | ***KIT*** | Gain | 22 | 16888899 | 51197838 | q11.1 | q13.33 | 544 genes |
| UPN163 | inv(16) | CBF2006 | ***NRAS, FLT3-TKD*** | Loss | 15 | 41966684 | 42103060 | q15.1 | q15.1 | *MGA, MIR626, MAPKBP1* |
|  |  |  |  | Gain | 22 | 16888899 | 51197838 | q11.1 | q13.33 | 544 genes |
| UPN165 | inv(16) | CBF2006 | ***WT1*** | Loss | 3 | 71194153 | 71558390 | p13 | p13 | *FOXP1* |
|  |  |  |  | CN-LOH | 19 | 28273327 | 42713835 | q11 | q13.2 | 286 genes |
|  |  |  |  | Loss | 19 | 42733538 | 44171480 | q13.2 | q13.31 | 41 genes |
|  |  |  |  | CN-LOH | 19 | 44193542 | 59097752 | q13.31 | q13.43 | 646 genes |
| UPN166 | inv(16) | CBF2006 | ***KIT*** | Gain | 12 | 32137342 | 32241827 | p11.21 | p11.21 | *KIAA1551, RNU6-78P* |
| UPN175 | inv(16) | CBF2006 | ***FLT3-TKD, BCORL1*** | CN-LOH | 2 | 15702 | 3994098 | p25.3 | p25.3 | 19 genes |
|  |  |  |  | CN-LOH | 5 | 146037036 | 175214075 | q32 | q35.2 | 178 genes |
|  |  |  |  | CN-LOH | 14 | 69786062 | 80408302 | q24.1 | q31.1 | 109 genes |
| UPN176 | inv(16) | CBF2006 | ***NRAS, FLT3-TKD, WT1*** | Gain | 8 | 158048 | 146295771 | p23.3 | q24.3 | 837 genes |
|  |  |  |  | Gain | 21 | 15006457 | 48097372 | q11.2 | q22.3 | 293 genes |
| UPN177 | inv(16) | CBF2006 | ***FLT3-ITD*** | Loss | 3 | 71111390 | 71523438 | p13 | p13 | *FOXP1* |
|  |  |  |  | Loss | 6 | 40950968 | 42190417 | p21.1 | p21.1 | 31 genes |
| UPN178 | inv(16) | CBF2006 | ***KIT, NRAS, FLT3-TKD*** | CN-LOH | 11 | 40143675 | 59932626 | p12 | q12.2 | 210 genes |
|  |  |  |  | CN-LOH | 14 | 70495006 | 88232559 | q24.2 | q31.3 | 111 genes |
|  |  |  |  | CN-LOH | 16 | 66126855 | 80563716 | q21 | q23.2 | 178 genes |
| UPN179 | inv(16) | CBF2006 | ***FLT3-TKD*** | Gain | 8 | 130505871 | 130742165 | q24.21 | q24.21 | *CCDC26* |
| UPN180 | inv(16) | CBF2006 | None | Gain | 21 | 15006457 | 48097372 | q11.2 | q22.3 | 293 genes |
| UPN181 | inv(16) | CBF2006 | ***WT1*** | Gain | 8 | 158048 | 146295771 | p23.3 | q24.3 | 837 genes |
|  |  |  |  | CN-LOH | 17 | 27093541 | 81041938 | q11.2 | q25.3 | 926 genes |
| UPN187 | inv(16) | CBF2006 | ***KIT*** | Loss | 16 | 15822262 | 16253779 | p13.11 | p13.11 | *MYH11, FOPNL, ABCC1, ABCC6* |
|  |  |  |  | Loss | 16 | 67131672 | 67373289 | q22.1 | q22.1 | 19 genes |
| UPN199 | inv(16) | CBF2006 | ***KIT, FLT3-TKD*** | Loss | 16 | 15810206 | 16099147 | p13.11 | p13.11 | *NDE1, MYH11, FOPNL, ABCC1* |
| UPN202 | inv(16) | CBF2006 | None | CN-LOH | 8 | 18654793 | 38835269 | p22 | p11.22 | 147 genes |
| UPN215 | inv(16) | CBF2006 | ***NRAS*** | Gain | 8 | 158048 | 146295771 | p23.3 | q24.3 | 837 genes |
|  |  |  |  | Gain | 22 | 16888899 | 51197838 | q11.1 | q13.33 | 544 genes |
| UPN300 | inv(16) | CBF2006 | None | Loss | 11 | 28720899 | 51581438 | p14.1 | p11.12 | 141 genes |
| UPN313 | inv(16) | CBF2006 | None | Loss | 1 | 62843769 | 63903245 | p31.3 | p31.3 | *USP1, DOCK7, ANGPTL3, ATG4C, LINC00466, FOXD3, ALG6* |
|  |  |  |  | Gain | 8 | 158048 | 146295771 | p23.3 | q24.3 | 837 genes |
|  |  |  |  | Loss | 16 | 15822262 | 16272796 | p13.11 | p13.11 | *MYH11, FOPNL, ABCC1, ABCC6* |
| UPN316 | inv(16) | CBF2006 | None | Loss | 3 | 71167808 | 71674038 | p13 | p13 | *FOXP1, MIR1284* |
|  |  |  |  | Gain | 22 | 16888899 | 51197838 | q11.1 | q13.33 | 542 genes |
| UPN323 | inv(16) | CBF2006 | None | Loss | 7 | 115428019 | 153419287 | q31.2 | q36.2 | 318 genes |
| UPN1 | t(8;21) | ELAM02 | ND | Loss | 1 | 76959912 | 81292223 | p31.1 | p31.1 | 17 genes |
|  |  |  |  | Loss | 16 | 49816368 | 59698177 | q12.1 | q21 | 99 genes |
|  |  |  |  | Loss | Y | 2650140 | 28799937 | p11.31 | q11.23 | 102 genes |
| UPN3 | t(8;21) | ELAM02 | ***KIT, ASXL1, KDM6A, RAD21*** | Loss | Y | 2650140 | 28799937 | p11.31 | q11.23 | 102 genes |
| UPN6 | t(8;21) | ELAM02 | ***KIT, ASXL2, RAD21, PHF6, WT1*** | Gain | 7 | 157867620 | 158385584 | q36.3 | q36.3 | *PTPRN2, MIR595, MIR5707* |
|  |  |  |  | CN-LOH | 18 | 72412442 | 78014582 | q22.3 | q23 | 25 genes |
|  |  |  |  | Loss | X | 168546 | 155233731 | p22.33 | q28 | 1015 genes |
| UPN10 | t(8;21) | ELAM02 | ***KIT*** | Gain | 8 | 158048 | 146295771 | p23.3 | q24.3 | 837 genes |
|  |  |  |  | Loss | X | 168546 | 155233731 | p22.33 | q28 | 1016 genes |
| UPN14 | t(8;21) | ELAM02 | ***KIT, SMC3, WT1*** | Loss | Y | 2650140 | 28799937 | p11.31 | q11.23 | 102 genes |
| UPN16 | t(8;21) | ELAM02 | ***KIT, NRAS, BCORL1*** | Loss | 3 | 121347763 | 176529424 | q13.33 | q26.32 | 347 genes |
|  |  |  |  | Gain | 20 | 61568 | 62915555 | p13 | q13.33 | 653 genes |
| UPN17 | t(8;21) | ELAM02 | ***IDH2*** | Loss | 9 | 70966261 | 95539740 | q21.11 | q22.31 | 129 genes |
|  |  |  |  | Loss | X | 168546 | 155233731 | p22.33 | q28 | 1020 genes |
| UPN18 | t(8;21) | ELAM02 | ***JAK2*** | CN-LOH | 6 | 89915403 | 114585822 | q15 | q21 | 103 genes |
|  |  |  |  | CN-LOH | 9 | 120517843 | 139593195 | q33.1 | q34.3 | 279 genes |
|  |  |  |  | CN-LOH | 10 | 95661 | 7069403 | p15.3 | p14 | 48 genes |
|  |  |  |  | CN-LOH | 10 | 30019506 | 68420793 | p11.23 | q21.3 | 176 genes |
|  |  |  |  | CN-LOH | 13 | 19438806 | 22562993 | q11 | q12.11 | 29 genes |
|  |  |  |  | CN-LOH | 17 | 77135790 | 81041938 | q25.3 | q25.3 | 94 genes |
|  |  |  |  | Loss | Y | 2650140 | 28799937 | p11.31 | q11.23 | 102 genes |
| UPN21 | t(8;21) | ELAM02 | ***TET2*** | Loss | 2 | 192692553 | 242783384 | q32.3 | q37.3 | 397 genes |
|  |  |  |  | Gain | 4 | 118037149 | 190957473 | q26 | q35.2 | 273 genes |
|  |  |  |  | Gain | 17 | 41544328 | 81041938 | q21.31 | q25.3 | 585 genes |
|  |  |  |  | CN-LOH | 19 | 260911 | 20482921 | p13.3 | p12 | 622 genes |
|  |  |  |  | Gain | Y | 2905395 | 3328404 | p11.31 | p11.2 | *LINC00278* |
| UPN22 | t(8;21) | ELAM02 | ***KIT, NRAS*** | Gain | 3 | 29978593 | 31092502 | p24.1 | p23 | *RBMS3, TGFBR2, GADL1* |
|  |  |  |  | Loss | Y | 2650140 | 28799937 | p11.31 | q11.23 | 102 genes |
| UPN23 | t(8;21) | ELAM02 | ***KIT, KRAS, RAD21*** | Gain | 8 | 158048 | 146295771 | p23.3 | q24.3 | 837 genes |
|  |  |  |  | Loss | X | 168546 | 155233731 | p22.33 | q28 | 1020 genes |
| UPN26 | t(8;21) | ELAM02 | ***NRAS, IDH2*** | Loss | 7 | 120300037 | 159119707 | q31.31 | q36.3 | 333 genes |
|  |  |  |  | Gain | X | 78292325 | 155233731 | q21.1 | q28 | 508 genes |
| UPN28 | t(8;21) | ELAM02 | ***SMC1A*** | Loss | X | 168546 | 155233731 | p22.33 | q28 | 1020 genes |
| UPN30 | t(8;21) | ELAM02 | ND | Loss | Y | 2650140 | 28799937 | p11.31 | q11.23 | 102 genes |
| UPN31 | t(8;21) | ELAM02 | ***NRAS*** | Loss | 7 | 118513314 | 159119707 | q31.31 | q36.3 | 333 genes |
|  |  |  |  | Loss | 8 | 80664947 | 81003919 | q21.13 | q21.13 | *HEY1, MRPS28, TPD52* |
|  |  |  |  | Gain | 13 | 83994294 | 115107733 | q31.1 | q34 | 137 genes |
| UPN34 | t(8;21) | ELAM02 | ***KIT, ASXL1*** | Loss | 2 | 198345796 | 220260561 | q33.1 | q35 | 182 genes |
|  |  |  |  | Loss | 8 | 93096598 | 93760245 | q21.3 | q22.1 | *RUNX1T1, FLJ46284* |
| UPN36 | t(8;21) | ELAM02 | ***NRAS*** | Gain | 8 | 158048 | 146295771 | p23.3 | q24.3 | 837 genes |
|  |  |  |  | Loss | X | 168546 | 155233731 | p22.33 | q28 | 1020 genes |
| UPN37 | t(8;21) | ELAM02 | ***KIT, NRAS, ASXL2*** | Loss | 9 | 71019572 | 108535812 | q21.11 | q31.2 | 244 genes |
|  |  |  |  | Gain | 18 | 6195413 | 6412607 | p11.31 | p11.31 | *L3MBTL4, MIR4317* |
|  |  |  |  | Gain | 18 | 8923060 | 9110151 | p11.22 | p11.22 | *NDUFV2* |
| UPN39 | t(8;21) | ELAM02 | ***NRAS, FLT3-ITD*** | Loss | 7 | 137264297 | 151938209 | q33 | q36.1 | 178 genes |
|  |  |  |  | Loss | X | 168546 | 155233731 | p22.33 | q28 | 1020 genes |
| UPN40 | t(8;21) | ELAM02 | ***KIT, FLT3-ITD, ASXL2*** | Loss | Y | 2650140 | 28799937 | p11.31 | q11.23 | 102 genes |
| UPN44 | t(8;21) | ELAM02 | ND | Loss | 9 | 74686623 | 109508320 | q21.13 | q31.2 | 220 genes |
| UPN45 | t(8;21) | ELAM02 | ***EZH2, IDH1*** | CN-LOH | 7 | 76449061 | 159119220 | q11.23 | q36.3 | 638 genes |
|  |  |  |  | Loss | X | 168546 | 155233731 | p22.33 | q28 | 1020 genes |
| UPN50 | t(8;21) | ELAM02 | ***KIT, NRAS, PTPN11, ASXL2*** | Loss | 8 | 93084517 | 93128271 | q21.3 | q21.3 | *RUNX1T1* |
| UPN51 | t(8;21) | ELAM02 | ***FLT3-TKD, PTPN11*** | Loss | 7 | 121676835 | 159119707 | q31.32 | q36.3 | 327 genes |
|  |  |  |  | Gain | 8 | 130501929 | 130742165 | q24.21 | q24.21 | *CCDC26* |
|  |  |  |  | Gain | 13 | 58483197 | 115107733 | q21.1 | q34 | 187 genes |
| UPN53 | t(8;21) | ELAM02 | ***KIT, FLT3-TKD, ASXL1, TET2*** | CN-LOH | 17 | 18562719 | 29861845 | p11.2 | q11.2 | 135 genes |
| UPN54 | t(8;21) | ELAM02 | ***KIT*** | Gain | 8 | 93084427 | 146295771 | q21.3 | q24.3 | 318 genes |
|  |  |  |  | Loss | 9 | 70999374 | 105036059 | q21.11 | q31.1 | 225 genes |
|  |  |  |  | Gain | 21 | 15318527 | 36219566 | q11.2 | q22.12 | 140 genes |
|  |  |  |  | Loss | Y | 2650140 | 28799937 | p11.31 | q11.23 | 102 genes |
| UPN55 | t(8;21) | ELAM02 | ***EZH2, GATA2*** | Loss | 7 | 147505608 | 159089304 | q35 | q36.3 | 107 genes |
|  |  |  |  | Loss | Y | 2650140 | 28799937 | p11.31 | q11.23 | 102 genes |
| UPN56 | t(8;21) | ELAM02 | ***KDM6A*** | Loss | Y | 2650140 | 28799937 | p11.31 | q11.23 | 102 genes |
| UPN58 | t(8;21) | ELAM02 | ND | Gain | 1 | 191670027 | 249224684 | q31.2 | q44 | 483 genes |
|  |  |  |  | Loss | 7 | 109073225 | 159089304 | q31.1 | q36.3 | 365 genes |
|  |  |  |  | Gain | 9 | 241329 | 38787480 | p24.3 | p13.1 | 235 genes |
|  |  |  |  | Loss | 9 | 67983173 | 119715132 | q13 | q33.1 | 340 genes |
|  |  |  |  | Gain | 9 | 119721743 | 141020389 | q33.1 | q34.3 | 352 genes |
| UPN60 | t(8;21) | ELAM02 | ***NRAS, ASXL2, SMC3*** | CN-LOH | 2 | 15702 | 5871954 | p25.3 | p25.2 | 22 genes |
|  |  |  |  | Gain | 21 | 36355481 | 36423085 | q22.12 | q22.12 | *RUNX1, RUNX1-IT1* |
| UPN63 | t(8;21) | ELAM02 | ND | Loss | 12 | 7137602 | 7183132 | p13.31 | p13.31 | *C1S* |
| UPN64 | t(8;21) | ELAM02 | ***KIT, EZH2*** | Loss | Y | 2650140 | 28799937 | p11.31 | q11.23 | 102 genes |
| UPN65 | t(8;21) | ELAM02 | ***KIT, ASXL2*** | Loss | 8 | 93096598 | 93866634 | q21.3 | q22.1 | *RUNX1T1, FLJ46284* |
| UPN68 | t(8;21) | ELAM02 | ***NRAS, FLT3-ITD*** | Gain | 1 | 227833996 | 249224684 | q42.13 | q44 | 197 genes |
| UPN71 | t(8;21) | ELAM02 | ***KIT*** | Gain | 4 | 52689101 | 190957473 | q11 | q35.2 | 613 genes |
|  |  |  |  | Loss | 7 | 145432939 | 158192663 | q35 | q36.3 | 104 genes |
| UPN72 | t(8;21) | ELAM02 | ***NRAS*** | Loss | Y | 2650140 | 28799937 | p11.31 | q11.23 | 102 genes |
| UPN73 | t(8;21) | ELAM02 | ***KIT, STAG2*** | Loss | 7 | 48524861 | 50457187 | p12.3 | p12.2 | *ABCA13, CDC14C, VWC2, ZPBP, C7orf72, IKZF1* |
|  |  |  |  | Loss | 9 | 69977403 | 120208344 | q21.11 | q33.1 | 333 genes |
| UPN75 | t(8;21) | CBF2006 | ***KIT, EZH2*** | Loss | 7 | 39655499 | 40318447 | p14.1 | p14.1 | *RALA, LINC00265, CDK13, MPLKIP, C7orf10* |
|  |  |  |  | Loss | Y | 2650140 | 28799937 | p11.31 | q11.23 | 102 genes |
| UPN81 | t(8;21) | CBF2006 | ***KIT, NRAS*** | Loss | 9 | 96875555 | 106020056 | q22.32 | q31.1 | 84 genes |
|  |  |  |  | Loss | 11 | 26172841 | 37092214 | p14.2 | p12 | 65 genes |
|  |  |  |  | Loss | Y | 2650140 | 28799937 | p11.31 | q11.23 | 102 genes |
| UPN85 | t(8;21) | CBF2006 | ND | Loss | X | 168546 | 155233731 | p22.33 | q28 | 1020 genes |
| UPN87 | t(8;21) | CBF2006 | ***KIT, ASXL1*** | Loss | 2 | 179566199 | 187541330 | q31.2 | q32.1 | 23 genes |
|  |  |  |  | Loss | 2 | 202901909 | 226360653 | q33.1 | q36.3 | 173 genes |
|  |  |  |  | Loss | 7 | 123365366 | 152984081 | q31.32 | q36.2 | 282 genes |
|  |  |  |  | CN-LOH | 11 | 44973081 | 69462856 | p11.2 | q13.3 | 496 genes |
| UPN92 | t(8;21) | CBF2006 | ***KIT, ASXL2*** | Loss | 9 | 70981817 | 88700253 | q21.11 | q21.33 | 71 genes |
|  |  |  |  | Gain | 12 | 109623177 | 109723974 | q24.11 | q24.11 | *ACACB, FOXN4* |
| UPN96 | t(8;21) | CBF2006 | ***KIT, FLT3-TKD, ASXL2, SMC3*** | Gain | 8 | 93083351 | 146295771 | q21.3 | q24.3 | 318 genes |
|  |  |  |  | Gain | 21 | 15006457 | 36223627 | q11.2 | q22.12 | 142 genes |
| UPN98 | t(8;21) | CBF2006 | ***ASXL1*** | Gain | 6 | 135432529 | 135691186 | q23.3 | q23.3 | *MYB, AHI1* |
|  |  |  |  | Loss | 8 | 117823216 | 118616883 | q24.11 | q24.11 | *RAD21, RAD21-AS1, MIR3610, AARD, SLC30A8, MED30* |
|  |  |  |  | Gain | 8 | 130538900 | 130789472 | q24.21 | q24.21 | *GSDMC, CCDC26* |
|  |  |  |  | Loss | Y | 2650140 | 28799937 | p11.31 | q11.23 | 102 genes |
| UPN104 | t(8;21) | CBF2006 | ND | Loss | 20 | 33419946 | 33929940 | q11.22 | q11.22 | 15 genes |
|  |  |  |  | Loss | Y | 2650140 | 28799937 | p11.31 | q11.23 | 102 genes |
| UPN107 | t(8;21) | CBF2006 | ***NRAS, KRAS*** | Gain | 8 | 130538900 | 130736854 | q24.21 | q24.21 | *CCDC26* |
| UPN109 | t(8;21) | CBF2006 | ***RAD21*** | Gain | 2 | 239324541 | 239451902 | q37.3 | q37.3 | *ASB1, LOC151171* |
|  |  |  |  | Gain | 11 | 88661443 | 89810326 | q14.3 | q14.3 | 13 genes |
|  |  |  |  | Loss | X | 168546 | 155233731 | p22.33 | q28 | 1019 genes |
| UPN114 | t(8;21) | CBF2006 | ***EZH2, TET2, SMC1A*** | Loss | 9 | 70996365 | 86951615 | q21.11 | q21.33 | 66 genes |
|  |  |  |  | Loss | 9 | 125331046 | 125487273 | q33.2 | q33.2 | *OR1Q1, OR1B1, OR1L1, OR1L3, OR1L4* |
|  |  |  |  | Loss | Y | 2650140 | 28799937 | p11.31 | q11.23 | 102 genes |
| UPN116 | t(8;21) | CBF2006 | ***ETV6*** | Loss | Y | 2650140 | 28799937 | p11.31 | q11.23 | 102 genes |
| UPN118 | t(8;21) | CBF2006 | ***KIT, FLT3-ITD*** | Loss | X | 168546 | 155233731 | p22.33 | q28 | 1020 genes |
| UPN125 | t(8;21) | CBF2006 | ***NRAS*** | CN-LOH | 19 | 17637160 | 32444501 | p13.11 | q13.11 | 131 genes |
| UPN126 | t(8;21) | CBF2006 | ***NRAS, RAD21*** | Gain | 14 | 81345865 | 81655518 | q31.1 | q31.1 | *CEP128, TSHR, GTF2A1* |
|  |  |  |  | Loss | Y | 2650140 | 28799937 | p11.31 | q11.23 | 102 genes |
| UPN128 | t(8;21) | CBF2006 | ***NRAS, FLT3-TKD*** | Loss | Y | 2650140 | 28799937 | p11.31 | q11.23 | 102 genes |
| UPN130 | t(8;21) | CBF2006 | ***KIT, ASXL1, KDM6A, TET2*** | CN-LOH | 4 | 54657395 | 190921709 | q12 | q35.2 | 595 genes |
|  |  |  |  | Loss | 21 | 36137571 | 36215159 | q22.12 | q22.12 | *LOC100506385, RUNX1* |
| UPN132 | t(8;21) | CBF2006 | ***KIT, EZH2, GATA2*** | Loss | 2 | 204563014 | 243089444 | q33.2 | q37.3 | 330 genes |
|  |  |  |  | Loss | 21 | 36138801 | 36221072 | q22.12 | q22.12 | *LOC100506385, RUNX1* |
|  |  |  |  | Loss | Y | 2650140 | 28799937 | p11.31 | q11.23 | 102 genes |
| UPN134 | t(8;21) | CBF2006 | ***KIT, SMC3*** | Loss | Y | 2650140 | 28799937 | p11.31 | q11.23 | 102 genes |
| UPN141 | t(8;21) | CBF2006 | ***SMC1A*** | Gain | 5 | 71531704 | 71634449 | q13.2 | q13.2 | *MRPS27, PTCD2* |
|  |  |  |  | Loss | Y | 2650140 | 28799937 | p11.31 | q11.23 | 102 genes |
| UPN142 | t(8;21) | CBF2006 | ND | Loss | 7 | 116427460 | 151908681 | q31.2 | q36.1 | 310 genes |
|  |  |  |  | Gain | 9 | 132430734 | 132558960 | q34.11 | q34.11 | *PRRX2, PTGES* |
|  |  |  |  | Loss | 13 | 69635981 | 70675528 | q21.33 | q21.33 | *KLHL1* |
| UPN145 | t(8;21) | CBF2006 | ***ASXL1*** | Loss | 2 | 204298490 | 242783384 | q33.2 | q37.3 | 328 genes |
|  |  |  |  | Gain | 3 | 133241105 | 197851986 | q22.1 | q29 | 406 genes |
|  |  |  |  | CN-LOH | 19 | 260911 | 6535498 | p13.3 | p13.3 | 209 genes |
|  |  |  |  | Loss | Y | 2650140 | 28799937 | p11.31 | q11.23 | 102 genes |
| UPN148 | t(8;21) | CBF2006 | ***ASXL2*** | Loss | 9 | 71035702 | 90310975 | q21.11 | q21.33 | 80 genes |
| UPN149 | t(8;21) | CBF2006 | ***KIT, KRAS, PTPN11, IKZF1*** | Loss | X | 168546 | 155233731 | p22.33 | q28 | 1020 genes |
| UPN151 | t(8;21) | CBF2006 | ***KIT, FLT3-ITD, ASXL2*** | Loss | Y | 2650140 | 28799937 | p11.31 | q11.23 | 102 genes |
| UPN156 | t(8;21) | CBF2006 | ***FLT3-ITD*** | Loss | Y | 2650140 | 28799937 | p11.31 | q11.23 | 102 genes |
| UPN157 | t(8;21) | CBF2006 | ND | Loss | 7 | 136483469 | 153966260 | q33 | q36.2 | 187 genes |
|  |  |  |  | Gain | 8 | 158048 | 146295771 | p23.3 | q24.3 | 837 genes |
|  |  |  |  | Loss | X | 168546 | 155233731 | p22.33 | q28 | 1020 genes |
| UPN158 | t(8;21) | CBF2006 | ***KDM6A, EZH2*** | CN-LOH | 10 | 95661 | 26722622 | p15.3 | p12.1 | 150 genes |
|  |  |  |  | Loss | X | 168546 | 155233731 | p22.33 | q28 | 1012 genes |
| UPN164 | t(8;21) | CBF2006 | ***ASXL1*** | Loss | 9 | 68734571 | 105787092 | q21.11 | q31.1 | 240 genes |
|  |  |  |  | Loss | X | 168546 | 155233731 | p22.33 | q28 | 1020 genes |
| UPN167 | t(8;21) | CBF2006 | ***ASXL2*** | Gain | 8 | 130586319 | 130736854 | q24.21 | q24.21 | *CCDC26* |
|  |  |  |  | Loss | Y | 2650140 | 28799937 | p11.31 | q11.23 | 102 genes |
| UPN168 | t(8;21) | CBF2006 | ***KIT, NRAS*** | Loss | Y | 2650140 | 28799937 | p11.31 | q11.23 | 102 genes |
| UPN169 | t(8;21) | CBF2006 | ND | Loss | 9 | 80806493 | 106878988 | q21.2 | q31.1 | 180 genes |
|  |  |  |  | Loss | Y | 2650140 | 28799937 | p11.31 | q11.23 | 102 genes |
| UPN170 | t(8;21) | CBF2006 | ***KIT*** | Loss | Y | 2650140 | 28799937 | p11.31 | q11.23 | 102 genes |
| UPN171 | t(8;21) | CBF2006 | ND | Loss | 5 | 95594812 | 96403620 | q15 | q15 | *PCSK1, CAST, ERAP1, ERAP2, LNPEP* |
|  |  |  |  | Loss | Y | 2650140 | 28799937 | p11.31 | q11.23 | 102 genes |
| UPN173 | t(8;21) | CBF2006 | ***KDM6A*** | CN-LOH | 19 | 260911 | 8596457 | p13.3 | p13.2 | 271 genes |
| UPN183 | t(8;21) | CBF2006 | ***KIT*** | Loss | 21 | 36183871 | 36210100 | q22.12 | q22.12 | *RUNX1* |
| UPN189 | t(8;21) | CBF2006 | ***FLT3-ITD*** | Loss | Y | 2650140 | 28799937 | p11.31 | q11.23 | 102 genes |
| UPN190 | t(8;21) | CBF2006 | ***KRAS, CBL, ASXL2*** | CN-LOH | 11 | 72919300 | 134942626 | q13.4 | q25 | 481 genes |
| UPN191 | t(8;21) | CBF2006 | ***KIT*** | Loss | Y | 2650140 | 28799937 | p11.31 | q11.23 | 102 genes |
| UPN192 | t(8;21) | CBF2006 | ***ASXL2*** | Loss | Y | 2650140 | 28799937 | p11.31 | q11.23 | 102 genes |
| UPN193 | t(8;21) | CBF2006 | ***KIT, ASXL2, RAD21*** | Loss | X | 145094628 | 145177681 | q27.3 | q27.3 | *MIR891A* |
| UPN197 | t(8;21) | CBF2006 | ND | Loss | 9 | 70966261 | 105396080 | q21.11 | q31.1 | 226 genes |
| UPN201 | t(8;21) | CBF2006 | ***NRAS, KRAS*** | Gain | 8 | 158048 | 93076586 | p23.3 | q21.3 | 520 genes |
|  |  |  |  | Loss | 8 | 93076600 | 93810560 | q21.3 | q22.1 | *RUNX1T1, FLJ46284* |
|  |  |  |  | Gain | 8 | 93810574 | 146295771 | q22.1 | q24.3 | 316 genes |
|  |  |  |  | Loss | 9 | 78067484 | 111660496 | q21.13 | q31.3 | 216 genes |
|  |  |  |  | Gain | X | 130719001 | 130967726 | q26.2 | q26.2 | *LOC286467* |
|  |  |  |  | Gain | Y | 16160763 | 16299733 | q11.221 | q11.221 | *VCY, VCY1B* |
| UPN203 | t(8;21) | CBF2006 | ***KIT, NRAS, ASXL1*** | Loss | X | 168546 | 155233731 | p22.33 | q28 | 1020 genes |
| UPN205 | t(8;21) | CBF2006 | ***NRAS, ASXL2*** | Gain | 8 | 158048 | 146295771 | p23.3 | q24.3 | 838 genes |
|  |  |  |  | Loss | X | 168546 | 155233731 | p22.33 | q28 | 1017 genes |
| UPN206 | t(8;21) | CBF2006 | ***KIT, ASXL1, SMC3*** | Loss | 9 | 70950015 | 105917090 | q21.11 | q31.1 | 227 genes |
|  |  |  |  | Loss | Y | 2650140 | 28799937 | p11.31 | q11.23 | 102 genes |
| UPN210 | t(8;21) | CBF2006 | ND | Loss | Y | 2650140 | 28799937 | p11.31 | q11.23 | 102 genes |
| UPN211 | t(8;21) | CBF2006 | ***NRAS, ASXL2*** | CN-LOH | 11 | 198509 | 34058107 | p15.5 | p13 | 398 genes |
| UPN214 | t(8;21) | CBF2006 | ***KIT, RUNX1*** | Loss | 8 | 93072679 | 93865548 | q21.3 | q22.1 | *RUNX1T1, FLJ46284* |
|  |  |  |  | Gain | 21 | 36219565 | 36469037 | q22.12 | q22.12 | *RUNX1, RUNX1-IT1* |
| UPN301 | t(8;21) | CBF2006 | ND | Loss | X | 168546 | 155233731 | p22.33 | q28 | 1017 genes |
| UPN302 | t(8;21) | CBF2006 | ND | Loss | X | 168546 | 155233731 | p22.33 | q28 | 1020 genes |
| UPN303 | t(8;21) | CBF2006 | ND | Loss | 8 | 115790446 | 117914100 | q23.3 | q24.11 | *TRPS1, LINC00536, EIF3H, UTP23, RAD21, RAD21-AS1, MIR3610* |
|  |  |  |  | Loss | 11 | 23254813 | 34833640 | p14.3 | p13 | 50 genes |
| UPN304 | t(8;21) | CBF2006 | ND | Gain | 8 | 130457109 | 130789472 | q24.21 | q24.21 | *GSDMC, CCDC26* |
|  |  |  |  | Gain | 11 | 117726648 | 117813237 | q23.3 | q23.3 | *FXYD6-FXYD2, FXYD6, TMPRSS13* |
|  |  |  |  | Loss | X | 168546 | 155233731 | p22.33 | q28 | 1016 genes |
| UPN309 | t(8;21) | CBF2006 | ND | Loss | Y | 2650140 | 28799937 | p11.31 | q11.23 | 102 genes |
| UPN310 | t(8;21) | CBF2006 | ND | Loss | 9 | 73171093 | 74433262 | q21.12 | q21.13 | *TRPM3, MIR204, TMEM2* |
|  |  |  |  | Loss | X | 168554 | 155230271 | p22.33 | q28 | 1020 genes |
| UPN311 | t(8;21) | CBF2006 | ND | CN-LOH | 8 | 128657530 | 146292734 | q24.21 | q24.3 | 152 genes |
| UPN314 | t(8;21) | CBF2006 | ND | Loss | 4 | 39902385 | 40203030 | p14 | p14 | *PDS5A, LOC344967, N4BP2, RHOH* |
|  |  |  |  | Loss | 12 | 11903925 | 12314050 | p13.2 | p13.2 | *ETV6, RNU6-19P, BCL2L14, MIR1244-1, MIR1244-2, MIR1244-3, LRP6* |
|  |  |  |  | Loss | Y | 2650140 | 28799937 | p11.31 | q11.23 | 102 genes |
| UPN315 | t(8;21) | CBF2006 | ND | Loss | 3 | 42557198 | 42610177 | p22.1 | p22.1 | *VIPR1, SEC22C* |
|  |  |  |  | Loss | Y | 2650140 | 28799937 | p11.31 | q11.23 | 102 genes |
| UPN317 | t(8;21) | CBF2006 | ND | Loss | 9 | 15159900 | 15351505 | p22.3 | p22.3 | *TTC39B* |
|  |  |  |  | Loss | X | 168546 | 155233731 | p22.33 | q28 | 1020 genes |
| UPN322 | t(8;21) | CBF2006 | ND | Loss | Y | 2650140 | 28799937 | p11.31 | q11.23 | 102 genes |
| UPN5 | inv(16) | ELAM02 | ***KIT*** | No SNP-array lesion | | | | | | |
| UPN8 | inv(16) | ELAM02 | ***KIT, KRAS*** | No SNP-array lesion | | | | | | |
| UPN9 | inv(16) | ELAM02 | ***KIT*** | No SNP-array lesion | | | | | | |
| UPN25 | inv(16) | ELAM02 | ***KIT*** | No SNP-array lesion | | | | | | |
| UPN32 | inv(16) | ELAM02 | ***KIT, KRAS, BCORL1*** | No SNP-array lesion | | | | | | |
| UPN43 | inv(16) | ELAM02 | ***NRAS*** | No SNP-array lesion | | | | | | |
| UPN46 | inv(16) | ELAM02 | ***NRAS*** | No SNP-array lesion | | | | | | |
| UPN52 | inv(16) | ELAM02 | ***KIT, KRAS, FLT3-TKD*** | No SNP-array lesion | | | | | | |
| UPN69 | inv(16) | ELAM02 | ***KIT*** | No SNP-array lesion | | | | | | |
| UPN89 | inv(16) | CBF2006 | ***NRAS, KRAS, FLT3-TKD*** | No SNP-array lesion | | | | | | |
| UPN100 | inv(16) | CBF2006 | ***KRAS, BCOR*** | No SNP-array lesion | | | | | | |
| UPN101 | inv(16) | CBF2006 | ***KIT, FLT3-TKD*** | No SNP-array lesion | | | | | | |
| UPN105 | inv(16) | CBF2006 | ***NRAS*** | No SNP-array lesion | | | | | | |
| UPN110 | inv(16) | CBF2006 | ***KIT, NRAS, KRAS, PTPN11*** | No SNP-array lesion | | | | | | |
| UPN112 | inv(16) | CBF2006 |  | No SNP-array lesion | | | | | | |
| UPN117 | inv(16) | CBF2006 | ***NRAS, KRAS*** | No SNP-array lesion | | | | | | |
| UPN121 | inv(16) | CBF2006 | ***KRAS*** | No SNP-array lesion | | | | | | |
| UPN124 | inv(16) | CBF2006 | ***KIT, KRAS, FLT3-TKD*** | No SNP-array lesion | | | | | | |
| UPN159 | inv(16) | CBF2006 | ***NRAS, KRAS, FLT3-TKD*** | No SNP-array lesion | | | | | | |
| UPN184 | inv(16) | CBF2006 | ***KIT*** | No SNP-array lesion | | | | | | |
| UPN185 | inv(16) | CBF2006 | ***PTPN11*** | No SNP-array lesion | | | | | | |
| UPN194 | inv(16) | CBF2006 | ***NRAS*** | No SNP-array lesion | | | | | | |
| UPN198 | inv(16) | CBF2006 | ***NRAS*** | No SNP-array lesion | | | | | | |
| UPN213 | inv(16) | CBF2006 | ***NRAS*** | No SNP-array lesion | | | | | | |
| UPN306 | inv(16) | CBF2006 | ND | No SNP-array lesion | | | | | | |
| UPN318 | inv(16) | CBF2006 | ND | No SNP-array lesion | | | | | | |
| UPN319 | inv(16) | CBF2006 | ND | No SNP-array lesion | | | | | | |
| UPN13 | t(8;21) | ELAM02 | ***SMC1A*** | No SNP-array lesion | | | | | | |
| UPN33 | t(8;21) | ELAM02 | ***NRAS, FLT3-ITD, ASXL2, WT1*** | No SNP-array lesion | | | | | | |
| UPN42 | t(8;21) | ELAM02 | ***KIT, NRAS, KRAS, ASXL2, BCOR*** | No SNP-array lesion | | | | | | |
| UPN49 | t(8;21) | ELAM02 | ***KIT, WT1*** | No SNP-array lesion | | | | | | |
| UPN59 | t(8;21) | ELAM02 | ***JAK2*** | No SNP-array lesion | | | | | | |
| UPN67 | t(8;21) | ELAM02 | ***FLT3-ITD, ASXL2*** | No SNP-array lesion | | | | | | |
| UPN78 | t(8;21) | CBF2006 | ***ASXL2*** | No SNP-array lesion | | | | | | |
| UPN79 | t(8;21) | CBF2006 | ***NRAS*** | No SNP-array lesion | | | | | | |
| UPN80 | t(8;21) | CBF2006 | ***KIT, ASXL2, BCORL1*** | No SNP-array lesion | | | | | | |
| UPN95 | t(8;21) | CBF2006 | ***RAD21*** | No SNP-array lesion | | | | | | |
| UPN143 | t(8;21) | CBF2006 |  | No SNP-array lesion | | | | | | |
| UPN152 | t(8;21) | CBF2006 | ***KIT, KDM6A, RAD21*** | No SNP-array lesion | | | | | | |
| UPN153 | t(8;21) | CBF2006 | ***FLT3-ITD, ASXL2*** | No SNP-array lesion | | | | | | |
| UPN174 | t(8;21) | CBF2006 | None | No SNP-array lesion | | | | | | |
| UPN196 | t(8;21) | CBF2006 | None | No SNP-array lesion | | | | | | |
| UPN307 | t(8;21) | CBF2006 | ND | No SNP-array lesion | | | | | | |
| UPN308 | t(8;21) | CBF2006 | ND | No SNP-array lesion | | | | | | |
| UPN320 | t(8;21) | CBF2006 | ND | No SNP-array lesion | | | | | | |
| UPN321 | t(8;21) | CBF2006 | ND | No SNP-array lesion | | | | | | |

###

Identified copy number alterations (CNAs) and copy-neutral losses of heterozygosity (CN-LOH) are listed for each CBF AML case (n=198) with chromosome location, start and end positions according to human genome version 19 (hg19) and gene count (gene symbols are given if < 10). Mutational profiling from reference[1] is also reported. ND: not determined.
